# Dietary Intake Modifies the Association between Air Pollution and Chronic Diseases: A Systematic Review and Meta-analysis

**DOI:** 10.1016/j.advnut.2026.100709

**Published:** 2026-07-20

**Authors:** Ye Chen, Bangqin Cai, Fei Wang, Hongyu Xuan, Yongqiang Sun, Yang Xia, Yuhong Zhao, Lishen Shan, Hehua Zhang

**Affiliations:** 1 Department of Clinical Epidemiology, Shengjing Hospital of China Medical University, Shenyang, China; 2 Liaoning Key Laboratory of Precision Medical Research on Major Chronic Disease, Shengjing Hospital of China Medical Unviersity, Shenyang, China; 3 Department of Pediatrics, Shengjing Hospital of China Medical University, Shenyang, China; 4 Neusoft Corporation, Shenyang, China; 5 Clinical Trials and Translation Center, Shengjing Hospital of China Medical University, Shenyang, China

**Keywords:** air pollution, dietary factor, modification, particulate matter, chronic diseases, nitrogen oxides, meta-analysis

## Abstract

Interaction effects between dietary factors and air pollutants remain inconsistent. This systematic review and meta-analysis examined whether dietary intake modifies the air pollution–chronic disease risk association. This study searched PubMed, Embase, Web of Science, and the Cochrane from inception to May 2026. A total of 51 observational studies (cohort, case-control, and cross-sectional), comprising 9,956,549 participants, that examined the modifying effect of dietary intake on the association between air pollution exposure and chronic disease risk through stratified analyses were included. Stratum-specific pooled odds ratios (pORs) per 5 μg/m^3^ pollutant increase were estimated using random-effects models, whereas effect modification was assessed using deft analysis by pooling study-specific within-study interaction estimates. Particulate matter (PM) increased cardiovascular disease risk by 7%, attenuated by greater adherence to healthy dietary patterns (HDPs, *P*-int < 0.001, high compared with low intake pOR: 1.05 compared with 1.11), grain (*P*-int < 0.001, pOR: 1.04 compared with 1.06), and dairy (*P*-int < 0.001, pOR: 1.03 compared with 1.08), and amplified by sugar-sweetened beverage (SSB). PM and nitrogen oxides (NO_X_) increased chronic respiratory disease risk by 20% and 7%, respectively, attenuated by omega-3 (*n*–3) fatty acids (*P*-int = 0.002, pOR 1.05 compared with 1.09), and amplified by unhealthy dietary patterns (UDPs) and ω-6 fatty acids. PM and NO_X_ increased metabolic disorder risk by 19% and 13%, respectively, attenuated by HDPs (PM: *P*-int < 0.001, pOR 1.04 compared with 1.24; NO_X_: *P*-int < 0.001, pOR 1.01 compared with 1.07), and amplified by UDPs, SSB, and sodium. PM increased neuropsychiatric and digestive disease risk by 9% and 17%, respectively, attenuated by vitamin B (dementia: *P*-int < 0.001, pOR: 0.95 compared with 1.18). These findings suggest that greater adherence to HDPs, grain, dairy, ω-3 fatty acids, and vitamin B may attenuate the association between PM and NO_X_ exposure and chronic disease risk. This review was registered in PROSPERO as CRD420251148066.

## Introduction

**Statement of Significance**

This systematic review and meta-analysis is the first quantitative review to assess interaction effects, offering potential dietary prevention strategies to mitigate the harmful impact of air pollution on public health at 3 levels: dietary patterns, food items, and dietary nutrients.

Chronic diseases, defined as conditions lasting ≥1 y that require ongoing medical management or result in limitations in daily activities [1], account for nearly two-thirds of global disability-adjusted life years. Cardiovascular diseases (CVDs) (e.g., ischemic heart disease and stroke), cancers, chronic respiratory diseases (CRDs) [e.g., chronic obstructive pulmonary disease (COPD) and asthma], metabolic disorders (e.g., obesity and diabetes mellitus), neuropsychiatric disorders (e.g., depression and dementia), and digestive diseases (e.g., Crohn’s disease) are common chronic diseases that constitute a significant global health burden and underline the urgent need for prevention [2,3].

Ambient particulate matter (PM) air pollution is the second leading risk factor for total disease burden worldwide, as measured by disability-adjusted life years [2]. Both acute and chronic exposure to air pollution increase morbidity and mortality across a range of chronic diseases, including CVD, cancer, CRD, metabolic disorders, neuropsychiatric disorders, and digestive diseases [[4], [5], [6], [7], [8], [9], [10], [11], [12]]. Mechanistically, inhaled pollutants trigger primary responses such as oxidative stress, inflammation, autonomic dysregulation, and direct particle translocation [13,14]. In the cardiovascular system, these primary responses initiate secondary cascades—including endothelial dysfunction, thrombosis, hypothalamic–pituitary–adrenal axis overactivation, and epigenetic changes—that elevate CVD risk [15,16]. In the respiratory tract, pollutant-induced inflammation and epithelial injury promote COPD and asthma [17]. In metabolic tissues, impaired insulin signaling and dysregulated lipid metabolism increase susceptibility to obesity and type 2 diabetes [18]. Moreover, systemic and neural inflammation increase risk of neuropsychiatric disorders and cognitive decline [19], whereas gut inflammation and oxidative stress may contribute to digestive diseases [20]. Given the ubiquity of air pollutants, effective measures to reduce personal exposure remain limited, emphasizing the urgent need for individualized intervention strategies to mitigate the adverse effects of air pollution on chronic diseases.

Dietary intake, encompassing dietary patterns, food items, and specific nutrients, is a major modifiable contributor to the burden of leading chronic diseases, including CVD, type 2 diabetes, and site-specific cancers [[21], [22], [23]]. Adherence to healthy dietary patterns (HDPs) such as the Mediterranean diet (MED) and Dietary Approaches to Stop Hypertension diet; greater adherence to fruits, vegetables, whole grains, and nuts; and adequate consumption of antioxidants, PUFAs, and vitamins have all been associated with reduced proinflammatory biomarkers and a lower incidence of CVD, CRD, metabolic disorders, neuropsychiatric disorders, and digestive diseases [[24], [25], [26]]. Mechanistically, healthy dietary intake can reduce risk of chronic diseases by lowering systemic inflammation and oxidative stress, improving immune function, modulating the gut microbiota, and supporting metabolic and endothelial health [[27], [28], [29], [30]]. Because the reduction in chronic disease risk conferred by healthy dietary intake and the elevation induced by air pollution exposure are mediated by overlapping molecular pathways, dietary intake has the potential to act as an effect modifier of the air pollution–chronic disease risk association.

Previous studies have indicated that dietary intake may modify the impact of pollution on most chronic diseases [[31], [32], [33], [34], [35], [36]]. However, the findings are inconsistent; some studies have reported that healthy dietary factors [e.g., MED, whole grain, and omega-3 (*n*–3) fatty acids] can mitigate the negative impact of pollution on chronic diseases [[31], [32], [33],^36^], whereas others have not demonstrated a significant modification [34,35]. Therefore, the aim of this study was to systematically review and meta-analyze the evidence on whether dietary factors modify the association between air pollution and chronic disease risk and to identify possible dietary patterns, specific food items, and nutrients that may attenuate or exacerbate these effects, with the goal of providing evidence-based dietary strategies to reduce the health hazards of air pollution.

## Methods

This study was prospectively registered in PROSPERO (CRD420251148066) and followed the 2020 PRISMA reporting guidelines [37]. Two independent investigators (Y.C. and B.C.) performed the bibliographic search, study screening, quality evaluation, and information extraction. Discordant assessments were resolved through discussion with a third reviewer (H.Z.).

### Search strategy

This study systematically searched the PubMed, Embase, Web of Science, and Cochrane Library databases from inception to May 2026. The complete search strategy is presented in Supplemental Table 1. In addition, the citation lists of all eligible investigations and relevant reviews were manually examined to prevent omissions.

### Eligibility criteria

According to the Population, Exposure, Comparison, Outcome, Study design model [38], the inclusion criteria were as follows: *1*) Population: people of all ages; *2*) Exposure factors: dietary factors (dietary patterns, food items, or dietary nutrients) and ambient air pollution [PM with an aerodynamic diameter of ≤1 μm (PM_1_), ≤2.5 μm (PM_2.5_), ≤10 μm (PM_10_), or between 2.5 μm and 10 μm (PM_2.5-10_); nitrogen dioxide (NO_2_), and nitrogen oxides (NO_X_), which are collectively referred to as NO_X_; sulfur dioxide (SO_2_); carbon monoxide (CO); or ozone (O_3_)]; *3*) Outcome: any chronic disease; in accordance with the International Statistical Classification of Diseases and Related Health Problems, 10th Revision (ICD-10) [39], chronic diseases were systematically classified as CVD (ICD-10: I00-I99), CRD (ICD-10: J30-J99), metabolic disorders (ICD-10: E00-E90), neuropsychiatric disorders (ICD-10: F00-F99 and G00-G99), and digestive diseases (ICD-10: K00-K93). Cancers were classified into various systemic diseases based on their primary anatomical sites [40,41]. Details of the diseases included in this study can be found in Supplemental Table 2. *4*) Study design: cohort studies, cross-sectional studies, or case-control studies. *5*) Studies that conducted stratified analyses by dietary factors of the association between air pollution and risk of chronic diseases, with or without assessment of interaction effects.

The exclusion criteria were as follows: *1*) studies that did not report stratified analyses of dietary intake; *2*) studies in which the outcome did not include chronic diseases; *3*) nonoriginal research publications, such as reviews, commentaries, editorials, government reports, letters, animal and experimental studies, or conference abstracts; *4*) studies in which the study population was not a natural population (i.e., the control group did not consist of healthy individuals without major chronic diseases); *5*) non–English-language publications; and *6*) publications lacking requisite statistical parameters [effect estimates, 95% confidence intervals (CIs), and necessary data for their derivation].

### Data extraction

The following information was extracted: author, publication year, study design, follow-up time (applicable only to prospective cohort studies), sample size, age group, country, dietary intake, outcome, air pollution exposure, effect sizes (stratified effect estimates and 95% CIs, with or without *P* values for interaction effects), and adjusted covariates. For each study, stratum-specific effect estimates describing the association between air pollution exposure and chronic disease risk were extracted separately for the high- and low-dietary intake groups. Specifically, dietary patterns in which the main contributing foods recognized as healthy were grouped into the HDP group. In contrast, dietary patterns with main contributing foods considered detrimental to health were categorized as unhealthy dietary patterns (UDPs).

### Risk of bias and quality assessment

The modified Newcastle-Ottawa Scale [42] scoring criteria were used to evaluate the quality of the cohort and case-control studies, with scores of 0 to 3, 4 to 6, and 7 to9 representing low, medium, and high quality, respectively. The quality of the cross-sectional studies was evaluated using the Agency for Healthcare Research and Quality [43] scoring criteria, with scores of 0 to 3, 4 to 7, and 8 to 11 representing low, medium, and high quality, respectively.

### Statistical analysis

Stratified meta-analyses were performed for each dietary subgroup when a minimum of 3 independent effect estimates were available within each stratum. Odds ratios (ORs), risk ratios, and hazard ratios were considered equivalent measures of relative risk and pooled together [44]. For studies reporting standardized mean differences, ORs were estimated using the approximation method proposed by Chinn [45]. When effect estimates were reported as percent changes, log transformation was applied to approximate log ORs before back-transformation. All effect estimates were standardized to a 5 μg/m^3^ increment in air pollutant concentration whenever possible [46]. To facilitate the interpretation of the association between air pollution and chronic disease within dietary strata, pooled odds ratios (pORs) and the corresponding 95% CIs were estimated separately for high- and low-dietary intake groups using random-effects models.

Consistent with recommendations from the Cochrane Handbook [47] and the meta-analysis interaction approach described by Fisher et al. [48], the modifying effect of dietary intake was formally evaluated using the deft approach [48]. For each study, a study-specific interaction estimate was calculated as the difference between the log-transformed effect estimates in the high and low-dietary intake strata, corresponding to the log ratio of ORs (log ROR): log (ROR) = log (OR_high_) – log (OR_low_). The variance of each interaction estimate was derived from the reported 95% CIs of the stratum-specific effect estimates. Study-specific interaction estimates were subsequently pooled using a random-effects model to obtain the pooled within-study interaction effect (deft estimate), which was expressed as the pooled log ROR (95% CI) and the corresponding interaction *P* value (*P*-int). A significant modification effect of a given dietary factor was defined as *P*-int < 0.05. A positive and statistically significant pooled log ROR reflected a strengthening effect of dietary intake on the air pollution–outcome association, whereas a negative and significant value reflected a weakening effect.

Considering the potential differences in dietary intake patterns and susceptibility to air pollution between adults and children, separate meta-analyses were conducted when sufficient data were available. All analyses were performed using R software (version 4.5.2; R Core Team, 2025). Statistical significance was defined as a 2-sided *P* < 0.05.

## Results

### Study characteristics

**FIGURE 1:**
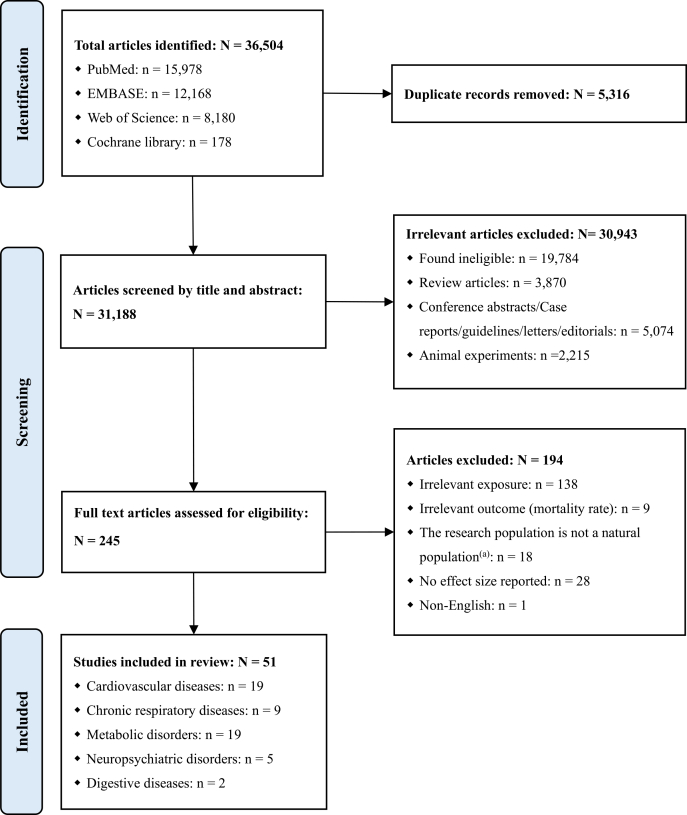
PRISMA flow diagram of the study selection and screening process. (A) Definition of natural population: control groups in included observational studies comprised healthy individuals free of major chronic diseases.

A total of 51 studies [[31], [32], [33], [34], [35], [36],[49], [50], [51], [52], [53], [54], [55], [56], [57], [58], [59], [60], [61], [62], [63], [64], [65], [66], [67], [68], [69], [70], [71], [72], [73], [74], [75], [76], [77], [78], [79], [80], [81], [82], [83], [84], [85], [86], [87], [88], [89], [90], [91], [92], [93]] involving 9,956,549 participants were included. The study selection workflow is shown in Figure 1. The included studies comprised 30 cohort studies, 1 case-control study, and 20 cross-sectional studies. The disease outcomes included CVD (19 studies), CRD (9 studies), metabolic disorders (19 studies), neuropsychiatric disorders (5 studies), and digestive diseases (2 studies). Air pollutant exposures included PM (47 studies), NO_X_ (22 studies), SO_2_ (8 studies), CO (3 studies), and O_3_ (6 studies). Dietary exposure was assessed based on nutrient intake (19 studies), dietary patterns (22 studies), and individual food intake (19 studies). The study populations were recruited from Europe (19 studies), Asia (28 studies), and the Americas (4 studies). The full details of the included studies are presented in Supplemental Table 3, and dietary exposure by disease system is listed in Supplemental Table 4.

### Quality assessment

All 51 included studies underwent risk of bias assessment, with 41 rated as high quality and 10 rated as moderate quality. The detailed risk of bias results for the cohort, case-control, and cross-sectional studies are provided in Supplemental Tables 5 to 7.

### Analysis of CVD

**FIGURE 2:**
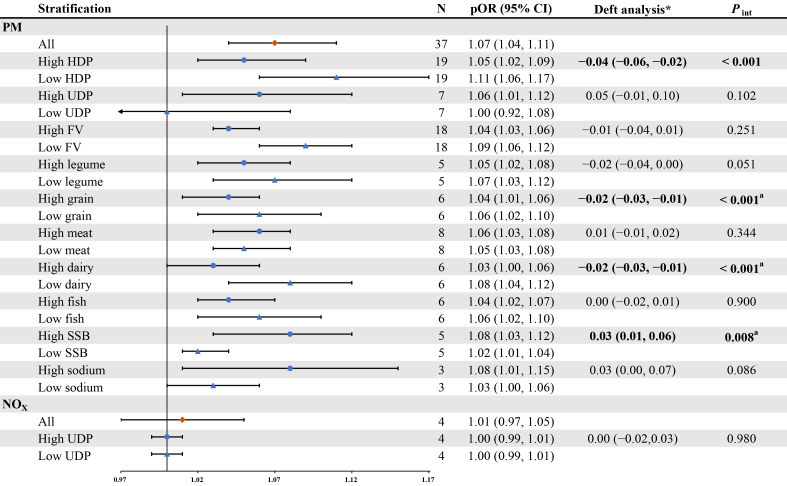
The modification of dietary intake on the association between air pollution and cardiovascular disease risk. ∗Deft analysis represents the pooled within-study interaction effect, expressed as the meta-analyzed log ratio of odds ratios with corresponding 95% confidence intervals; superscript a indicates that only hypertension (a single cardiovascular disease outcome) was included in the corresponding meta-analysis. CI, confidence interval; FV, fruits and vegetables; HDP, healthy dietary patterns; *N*, number of independent effect estimates included in the meta-analysis; NO_X_, nitrogen oxides; PM, particulate matter; pOR, pooled odds ratio; *P*-int, *P* value for interaction; SSB, sugar-sweetened beverages; UDP, unhealthy dietary patterns.

As shown in Figure 2, each 5 μg/m^3^ increase in PM concentration was associated with a 7% higher risk of CVD. This association was significantly attenuated by greater adherence to HDPs [*P*-int < 0.001; high compared with low intake: pOR 1.05 (95% CI: 1.02, 1.09) compared with 1.11 (1.06, 1.17)], grain intake [hypertension: *P*-int < 0.001; high compared with low intake: pOR 1.04 (95% CI: 1.01, 1.06) compared with 1.06 (1.02, 1.10)], and dairy intake [hypertension: *P*-int < 0.001; 1.03 (1.00, 1.06) compared with 1.08 (1.04, 1.12)] and significantly amplified by greater adherence to sugar-sweetened beverage (SSB) intake [hypertension: *P*-int = 0.008; 1.08 (1.03, 1.12) compared with 1.02 (1.01, 1.04)].

### Analysis of CRD

**FIGURE 3:**
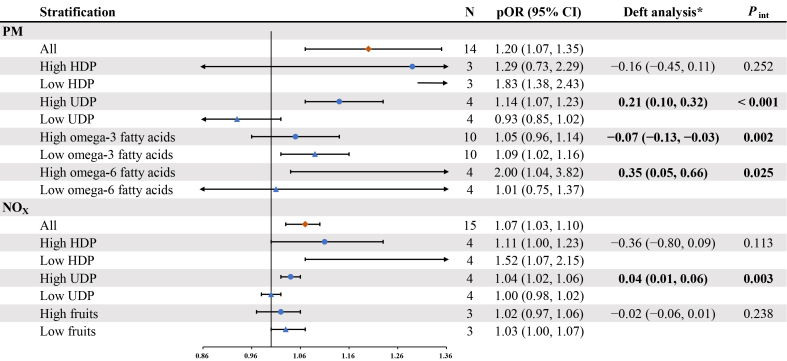
The modification of dietary intake on the association between air pollution and chronic respiratory disease risk. ∗Deft analysis represents the pooled within-study interaction effect, expressed as the meta-analyzed log ratio of odds ratios with corresponding 95% confidence intervals. CI, confidence interval; HDP, healthy dietary patterns; *N*, number of independent effect estimates included in the meta-analysis; NO_X_, nitrogen oxides; PM, particulate matter; pOR, pooled odds ratio; *P*-int, *P* value for interaction; UDP, unhealthy dietary patterns.

As shown in Figure 3, each 5 μg/m^3^ increase in PM and NO_X_ was associated with 20% and 7% higher risks of CRD, respectively. These adverse associations were significantly attenuated by greater adherence to ω-3 fatty acids [PM: *P*-int = 0.002; high compared with low intake: pOR 1.05 (0.96, 1.14) compared with 1.09 (1.02, 1.16)]. Conversely, they were significantly amplified by greater adherence to UDPs [PM: *P*-int < 0.001; pOR: 1.14 (1.07, 1.23) compared with 0.93 (0.85, 1.02); NO_X_: *P*-int = 0.003; 1.04 (1.02, 1.06) compared with 1.00 (0.98, 1.02)] and ω-6 fatty acids [*P*-int = 0.025; 2.00 (1.04, 3.82) compared with 1.01 (0.75, 1.37)].

### Analysis of metabolic disorders

**FIGURE 4:**
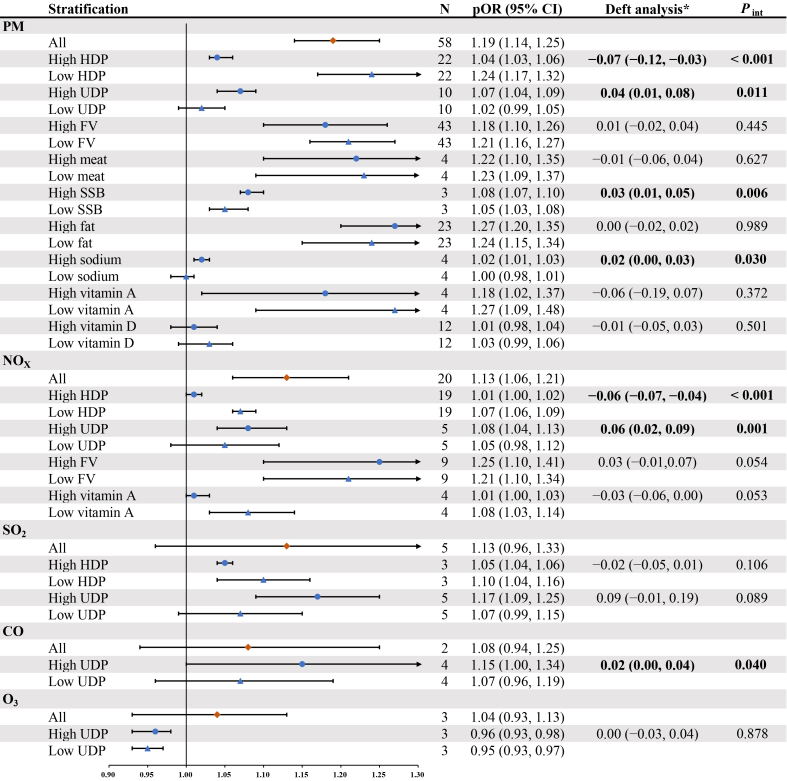
The modification of dietary intake on the association between air pollution and chronic metabolic system disease risk. ∗Deft analysis represents the pooled within-study interaction effect, expressed as the meta-analyzed log ratio of odds ratios with corresponding 95% confidence intervals. CI, confidence interval; FV, fruits and vegetables; HDP, healthy dietary patterns; N, number of independent effect estimates included in the meta-analysis; NO_X_, nitrogen oxides; PM, particulate matter; pOR, pooled odds ratio; *P*-int, *P* value for interaction; SSB, sugar-sweetened beverages; UDP, unhealthy dietary patterns.

As shown in Figure 4, each 5 μg/m^3^ increase in PM and NO_X_ was associated with 19% and 13% higher risks of metabolic disorders, respectively. These adverse associations were significantly attenuated by greater adherence to HDPs [PM: *P*-int < 0.001; high compared with low intake: pOR 1.04 (1.03, 1.06) compared with 1.24 (1.17, 1.32); NO_X_: *P*-int < 0.001; 1.01 (1.00, 1.02) compared with 1.07 (1.06, 1.09)]. Conversely, they were significantly amplified by greater adherence to UDPs [PM: *P*-int = 0.011; 1.07 (1.04, 1.09) compared with 1.02 (0.99, 1.05), NO_X_: *P*-int = 0.001; 1.08 (1.04, 1.13) compared with 1.05 (0.98, 1.12)], SSB intake [*P*-int = 0.006; 1.08 (1.07, 1.10) compared with 1.05 (1.03, 1.08)], and sodium intake [*P*-int = 0.030; 1.02 (1.01, 1.03) compared with 1.00 (0.98, 1.01)]. Although the overall association of CO with metabolic disorder risk was not statistically significant, greater adherence to UDPs significantly amplified this association [*P*-int = 0.040; 1.15 (1.00, 1.34) compared with 1.07 (0.96, 1.19)].

### Analysis of neuropsychiatric disorders and digestive diseases

**FIGURE 5:**
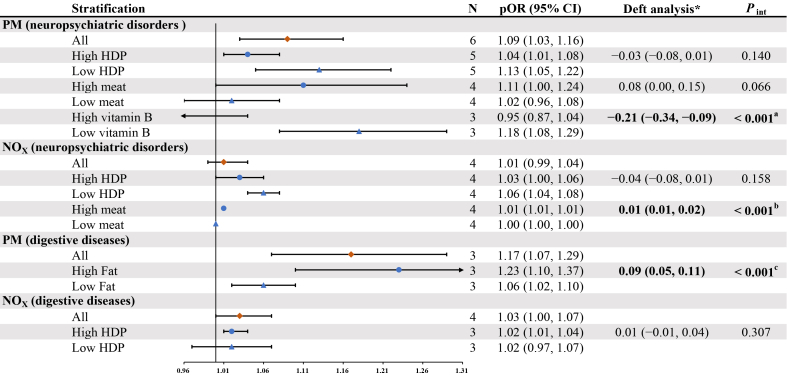
The modification of dietary intake on the association between air pollution and neuropsychiatric disorders and digestive disease risk. ∗Deft analysis represents the pooled within-study interaction effect, expressed as the meta-analyzed log ratio of odds ratios with corresponding 95% confidence intervals; Superscripts a, b, and c indicate that only dementia, depression, and metabolic-associated fatty liver disease, respectively, were included in the corresponding meta-analyses. CI, confidence interval; HDP, healthy dietary patterns; *N*, number of independent effect estimates included in the meta-analysis; NO_X_, nitrogen oxides; PM, particulate matter; pOR, pooled odds ratio; *P*-int, *P* value for interaction.

As shown in Figure 5, each 5 μg/m^3^ increase in PM was associated with 9% and 17% higher risks of neuropsychiatric disorders and digestive diseases, respectively. Greater adherence to vitamin B intake [dementia: *P*-int < 0.001; high compared with low: pOR 0.95 (0.87, 1.04) compared with 1.18 (1.08, 1.29)] attenuated this association, whereas fat intake strengthened the association with metabolic dysfunction-associated fatty liver disease (MAFLD) risk [*P*-int < 0.001; 1.23 (1.10, 1.37) compared with 1.06 (1.02, 1.10)]. Although the overall association of NO_X_ with neuropsychiatric disorder risk was not statistically significant, greater adherence to meat intake significantly amplified this association [depression: *P*-int < 0.001; 1.01 (1.01, 1.01) compared with 1.00 (1.00, 1.00)].

### Sensitivity analyses

Supplemental Figure 1 shows the results of the meta-analysis in children. Compared with the overall population, the modification by sodium [PM-metabolic disorders: *P*-int = 0.030; 1.02 (1.01, 1.03) compared with 1.00 (0.98, 1.01)] remained significant, whereas no modification by dairy products, grain, SSB, ω-3 fatty acids, or ω-6 fatty acids was observed. Supplemental Figure 2 shows the results for adults. Consistent with the overall population, the modification by grain [PM-CVD: *P*-int < 0.001; 1.03 (1.01, 1.06) compared with 1.06 (1.02, 1.10)], dairy products [PM-CVD: *P*-int < 0.001; 1.03 (0.99, 1.06) compared with 1.07 (1.02, 1.11)], SSB [PM-CVD: *P*-int = 0.008; 1.07 (1.03, 1.12) compared with 1.02 (1.01, 1.04)], and ω-3 fatty acids [PM-CRD: *P*-int = 0.002; 1.01 (0.94, 1.08) compared with 1.07 (1.00, 1.14)] remained significant, whereas no significant modification by ω-6 fatty acids was observed. Notably, a modest modifying effect of legume intake was observed among adults [PM-CVD: *P*-int = 0.048; 1.05 (1.02, 1.08) compared with 1.07 (1.03, 1.12)], although no significant effect modification was detected in the overall population. A few studies explored the interactions between other pollutants and dietary intake; however, <3 studies were available for any given exposure–outcome pair, precluding a meta-analysis (Supplemental Table 8 for details).

## Discussion

### Principal findings of this study

Through the systematic synthesis of stratified effect estimates from a large body of original studies, accompanied by within-study interaction analyses using the deft approach, this study found that HDPs attenuated the harmful effects of PM and NO_X_ on CVD and metabolic disorder risks, whereas UDPs amplified the impact of PM, NO_X_, and SO_2_ on CRD and metabolic disorder risks. Grain and dairy product intake reduced the effect of PM on hypertension, whereas SSB intake exacerbated the effects of PM on hypertension and risk of metabolic disorders. Meat intake exacerbated the effects of NO_X_ on depression. Among these nutrients, ω-3 fatty acids and vitamin B mitigated the effects of PM on CRD and dementia, respectively, whereas sodium, ω-6 fatty acids, and fat exacerbated the corresponding harmful effects. Notably, sodium exerted modifying effects primarily in children, whereas other dietary factors exerted modifying effects in adults.

### Dietary modulation of PM-associated CVD risk

Greater adherence to HDPs, grain intake, and dairy product intake reduced the adverse effects of PM on CVD, including heart failure, hypertension, stroke, and ventricular arrhythmias. In contrast, SSB intake increases the adverse effects of PM on CVD risk. A previous nonsystematic review similarly suggested that adherence to the MED may ameliorate the adverse health effects of air pollution; however, that review included only 2 primary studies on CVD mortality and provided no supporting quantitative synthesis [94]. Evidence from previous meta-analyses suggests that higher whole-grain consumption is associated with a reduced risk of hypertension, whereas refined-grain intake is not significantly associated with hypertension risk [95,96]. Additionally, 2 meta-analyses of prospective cohort studies reported that consumption of whole-fat and low-fat dairy products was associated with lower hypertension risk in adults [97,98]. Previous meta-analyses have consistently shown that higher consumption of SSB is associated with an increased risk of hypertension [[99], [100], [101]]. However, none of these studies have investigated the interaction between grain, dairy, and SSB intake and PM exposure. Sensitivity analyses did not replicate the modifying effects of grain, dairy, and SSB intake in children, which may reflect age-related physiological differences.

Collectively, HDPs, grains, and dairy products may act as effect modifiers of air pollution-related CVD risk in adults, driven by shared antioxidant and anti-inflammatory mechanisms. Bioactive compounds from HDPs improve endothelial function and inhibit free radical production and systemic inflammation, thereby alleviating PM-induced oxidative stress, inflammation, blood pressure elevation, autonomic dysfunction, and cardiovascular stress responses, ultimately reducing CVD risk [102]. Dairy bioactive compounds may exert parallel protection by mitigating PM-induced oxidative damage through antioxidant and anti-inflammatory pathways [103]. Because both SSB consumption and PM exposure promote oxidative stress, systemic inflammation, and endothelial dysfunction, high SSB intake may potentiate the hypertensive effects of PM via shared pathophysiological pathways [104].

### Dietary modulation of PM and NO_X_-associated CRD risk

This study found that ω-3 fatty acids mitigated the adverse effects of PM on risk of CRD (including COPD and lung cancer), whereas UDPs and ω-6 fatty acids exacerbated PM-related harm. Previous systematic reviews have linked UDPs to impaired lung function [105] and elevated lung cancer risk [106]. However, no studies have explored the interactions between dietary intake and air pollution. A previous review reported protective effects of ω-3 fatty acids against PM-induced damage [107]; however, it offered only a qualitative synthesis of randomized controlled trials, addressed symptom improvement rather than disease risk, and lacked quantitative data. Other reviews have found no significant association between ω-6 fatty acid intake and cancer or allergic disease risk [108,109] and have not investigated its interaction with PM. Sensitivity analyses further showed that the protective effects of ω-3 fatty acids were confined to adults (with COPD and lung cancer), likely because of the limited number of primary studies. Therefore, higher-quality original studies are needed to validate the effect modification of ω-3 and ω-6 fatty acids on air pollution-related CRD risk.

Mechanistically, ω-3 fatty acids may mitigate the adverse respiratory effects of PM by enhancing antioxidant defenses in airway epithelial cells and alveolar macrophages. This reduces local oxidative stress and NF-κB-mediated inflammatory cascades, thereby attenuating DNA damage, airway remodeling, and chronic lung inflammation, ultimately lowering COPD and lung cancer risk [[110], [111], [112]]. Conversely, proinflammatory dietary intake and ω-6 fatty acids may upregulate proinflammatory markers, including IL-6, amplify PM-induced airway injury, and synergistically increase risk of COPD progression, allergic rhinitis, and lung cancer [34,113,114].

### Dietary modulation of PM and NO_X_-associated metabolic disorder risk

HDPs mitigated the adverse effects of PM and NO_X_ on risk of metabolic disorders (including diabetes and metabolic syndrome), whereas SSB intake amplified the adverse effects of PM exposure on risk of metabolic syndrome. Previous systematic reviews have summarized the independent associations between HDPs and SSB intake and metabolic disorders, demonstrating that HDPs are associated with a lower risk of metabolic diseases [115,116] and that SSB intake is associated with a higher risk of metabolic syndrome [117,118]. However, the potential interactions between dietary intake and air pollution exposure have not been investigated. Further high-quality studies are required to verify the modification effects of HDPs and SSB. Mechanistically, HDPs may improve gut microbiota and lipid metabolism, enhance anti-inflammatory capacity, and alleviate insulin resistance and metabolic disturbances, thereby buffering risk of diabetes and metabolic syndrome associated with air pollution [119].

### Dietary modulation of PM-associated neuropsychiatric disorders and digestive disease risk

This study showed that vitamin B intake may attenuate the PM-related risk of dementia. A prior review indicated that B vitamins protect against PM-associated damage [107] and counteract PM-induced epigenetic modifications [120]; however, no quantitative meta-analysis was conducted. A high-fat diet amplified the effects of PM on MAFLD. A systematic review showed that high-fat dietary intake is positively associated with MAFLD risk [121]; however, no prior review has investigated fat–PM interactions. Mechanistically, PM activates hepatic Kupffer cells to initiate inflammation and oxidative DNA damage in the liver [122], whereas a high-fat diet exacerbates oxidative stress by altering fatty acid metabolism and lipid accumulation. Coexposure to PM and a high-fat diet promotes hepatic lipid deposition, inflammation, and hepatocellular injury. Given the limited number of original studies included, further high-quality investigations are warranted to verify these findings.

### Strengths and limitations

This study provides quantitative evidence for assessing interaction effects, offering potential dietary prevention strategies to mitigate the harmful impacts of air pollution on public health at 3 levels: dietary patterns, food items, and dietary nutrients. This study has certain limitations that must be acknowledged. First, substantial heterogeneity was observed in the meta-analysis, likely because of variability in study populations, geographic locations, disease outcomes, and inherent recall bias in dietary assessments. Second, research on CO, SO_2_, and O_3_ exposure, as well as on neuropsychiatric disorders and digestive disease outcomes, remains limited, which may affect the accuracy and comprehensiveness of the findings. Third, although sensitivity analyses were conducted separately for children and adults, the conclusions for these subgroups diverged from those for the overall population; because of the small number of studies, these results should be interpreted cautiously. Additionally, although the deft approach minimizes ecological bias by focusing on within-study interaction effects, the present analysis was based on published aggregate data rather than individual participant data; therefore, it may still be affected by residual confounding, inconsistent definitions of dietary strata, and limited reporting of stratum-specific effect estimates across studies.

### Implications

This meta-analysis suggests that dietary factors, including HDPs, grains, dairy products, ω-3 fatty acids, and vitamin B, may attenuate the adverse association between air pollution and risk of CVD, CRD, metabolic disorders, neuropsychiatric disorders, and digestive diseases. Future research should prioritize well-designed experimental studies and randomized controlled trials to further validate the effects of modifying dietary intake on the health impacts of air pollution and to elucidate the biological mechanisms underlying these modifying effects. Notably, current evidence regarding dietary modification primarily focuses on the adverse effects of PM and NO_X_. However, the potential modifying effects of dietary factors on other air pollutants require further investigation.

## Author contribution

The authors’ responsibilities were as follows – YC, BC: completion of interpretation of the data and the manuscript; FW, HX, YS: selection of literature and the extraction of data; YX, YZ: critical revision of the manuscript for important intellectual content; LS, HZ: concept, design, interpretation of the data, completion of the study and manuscript; and all authors: read and approved the final manuscript.

## Data availability

Data described in the manuscript and analytic code will be made available on proper application.

## Declaration of Generative AI and AI-assisted technologies in the writing process

The authors declare that no generative AI or AI-assisted technologies were used in the writing of this manuscript.

## Funding

This study was supported by the 345 Talent Project of Shengjing Hospital of China Medical University (grant number M0287 to Hehua Zhang) and the Natural Science Foundation of Liaoning Province (grant number 2024-MS-074 to Hehua Zhang). The sponsor contributed substantially to the concept, design, interpretation of the data, and completion of the study and manuscript. There are no restrictions regarding the submission or publication of this work.

## Conflict of interest

The authors report no conflicts of interest.

## References

[1] Serag H., Ghulmi L., Sallam H.S., Ferguson M., Manakatt B. (2024). Addressing chronic conditions and social determinants of health during the COVID-19 pandemic. Adv. Exp. Med. Biol..

[2] GBD 2023 Disease and Injury and Risk Factor Collaborators (2023). Burden of 375 diseases and injuries, risk-attributable burden of 88 risk factors, and healthy life expectancy in 204 countries and territories, including 660 subnational locations, 1990-2023: a systematic analysis for the Global Burden of Disease Study 2023. Lancet.

[3] GBD 2023 Causes of Death Collaborators (2023). Global burden of 292 causes of death in 204 countries and territories and 660 subnational locations, 1990-2023: a systematic analysis for the Global Burden of Disease Study 2023. Lancet.

[4] Nazar W., Niedoszytko M. (2022). Air pollution in Poland: a 2022 narrative review with focus on respiratory diseases. Int. J. Environ. Res. Public. Health..

[5] Dominski F.B.O.H., Lorenzetti Branco J.H., Buonanno G., Stabile L., Gameiro Da Silva M., Andrade A. (2021). Effects of air pollution on health: a mapping review of systematic reviews and meta-analyses. Environ. Res..

[6] Mannucci P.M., Harari S., Martinelli I., Franchini M. (2015). Effects on health of air pollution: a narrative review, Intern. Emerg. Med..

[7] Clifford A., Lang L., Chen R., Anstey K.J., Seaton A. (2016). Exposure to air pollution and cognitive functioning across the life course—a systematic literature review. Environ. Res..

[8] Borsi S.H., Goudarzi G., Sarizadeh G., Dastoorpoor M., Geravandi S., Shahriyari H.A. (2022). Health endpoint of exposure to criteria air pollutants in ambient air of on a populated in Ahvaz city, Iran, Front. Public. Health.

[9] Louis S., Carlson A.K., Suresh A., Rim J., Mays M., Ontaneda D. (2023). Impacts of climate change and air pollution on neurologic health, disease, and practice: a scoping review. Neurology.

[10] Luo H., Zhang Q., Yu K., Meng X., Kan H., Chen R. (2022). Long-term exposure to ambient air pollution is a risk factor for trajectory of cardiometabolic multimorbidity: a prospective study in the UK Biobank. EBioMedicine.

[11] Liang F., Liu F., Huang K., Yang X., Li J., Xiao Q. (2020). Long-term exposure to fine particulate matter and cardiovascular disease in China. J. Am. Coll. Cardiol..

[12] Kamarehei B., Farhadi M., Soleimani F., Dolati M., Sepahvand A., Bayat M. (2024). The level, source, and health outcome of PM_2.5_ exposure in Southwest Iran. Toxicol. Rep..

[13] Lee C.H., Chang M.H., Koh Y.H., Pack S.P., Seo M., Cha H. (2024). Mechanistic insight into airborne particulate matter PM_10_ as an environmental hazard for hemorrhagic stroke: evidence from in vitro and in vivo studies. J. Hazard. Mater..

[14] Li H., Guo X., Li P., Gao X., Song X., Chen X. (2024). Particulate matter induces depression-like behavior through systemic inflammation and brain-derived neurotrophic factors. Environ. Int..

[15] de Bont J., Jaganathan S., Dahlquist M., Persson A., Stafoggia M., Ljungman P. (2022). Ambient air pollution and cardiovascular diseases: an umbrella review of systematic reviews and meta-analyses. J. Intern. Med..

[16] Rajagopalan S., Al-Kindi S.G., Brook R.D. (2018). Air pollution and cardiovascular disease: JACC state-of-the-art review. J. Am. Coll. Cardiol..

[17] Sierra-Vargas M.P., Montero-Vargas J.M., Debray-García Y.N., Vizuet-de-Rueda J.C., Loaeza-Román A., Terán L.M. (2023). Oxidative stress and air pollution: its impact on chronic respiratory diseases. Int. J. Mol. Sci..

[18] Marchini T. (2023). Redox and inflammatory mechanisms linking air pollution particulate matter with cardiometabolic derangements. Free Radic. Biol. Med..

[19] Roy R., D’Angiulli A. (2024). Air pollution and neurological diseases, current state highlights. Front. Neurosci..

[20] Glencross D.A., Ho T.R., Camiña N., Hawrylowicz C.M., Pfeffer P.E. (2020). Air pollution and its effects on the immune system. Free Radic. Biol. Med..

[21] Wang M., Ma H., Qin C., Mandizadza O.O., Ni H., Shi Y. (2025). Burden of diet-related chronic diseases in Chinese and Japanese adults attributable to dietary risk factors from 1990 to 2021: a systematic analysis of the Global Burden of Disease Study 2021. Front. Nutr..

[22] An X., Liu Z., Zhang L., Zhao J., Gu Q., Han W. (2025). Co-occurrence patterns and related risk factors of ischaemic heart disease and ischaemic stroke across 203 countries and territories: a spatial correspondence and systematic analysis. Lancet Glob. Health.

[23] Ze W., Su R., Huang R., Guo Y., Yang X., Zhang B. (2025). A systematic analysis of the global disease burden of type 2 diabetes mellitus attributable to high intake of processed meat in 204 countries (1990-2021). Front. Endocrinol. (Lausanne)..

[24] Aleksandrova K., Koelman L., Rodrigues C.E. (2021). Dietary patterns and biomarkers of oxidative stress and inflammation: a systematic review of observational and intervention studies. Redox. Biol..

[25] Liu K.Q., Ding X.Y., Zhao W.H. (2020). Influence of dietary pattern on human immunity. Zhonghua Yi Xue Za Zhi..

[26] Keshani M., Rafiee S., Heidari H., Rouhani M.H., Sharma M., Bagherniya M. (2025). Mediterranean Diet reduces inflammation in adults: a systematic review and meta-analysis of randomized controlled trials. Nutr. Rev.

[27] Kelly F.J., Fussell J.C. (2015). Air pollution and public health: emerging hazards and improved understanding of risk. Environ. Geochem. Health..

[28] Rajagopalan S., Brook R.D., Salerno P.R.V.O., Bourges-Sevenier B., Landrigan P., Nieuwenhuijsen M.J. (2024). Air pollution exposure and cardiometabolic risk. Lancet. Diabetes. Endocrinol..

[29] Calder P.C. (2015). Marine omega-3 fatty acids and inflammatory processes: effects, mechanisms and clinical relevance. Biochim. Biophys. Acta..

[30] Xu X., Zhang J., Yang X., Zhang Y., Chen Z. (2020). The role and potential pathogenic mechanism of particulate matter in childhood asthma: a review and perspective. J. Immunol. Res..

[31] Huang K., Yu D., Fang H., Ju L., Piao W., Guo Q. (2023). Association of fine particulate matter and its constituents with hypertension: the modifying effect of dietary patterns. Environ. Health..

[32] Fan C., Wang W., Xiong W., Li Z., Ling L. (2025). Beverage consumption modifies the risk of type 2 diabetes associated with ambient air pollution exposure. Ecotoxicol. Environ. Saf..

[33] Wang T., Zhao C., Fang X., Zhao J., Chao W., Bo Y. (2025). Healthful plant-based dietary patterns associated with reduced adverse effects of air pollution on COPD: findings from a large cohort study. Nutrients.

[34] Yang Y.S., Tang X.W., Wu J.F., Zhan Z.Y., Hu Z.J., He F. (2025). Mitigating air pollution’s impact on lung cancer in a large-scale longitudinal study: the unexplored potential of dietary interventions. Ecotoxicol. Environ. Saf..

[35] Fan C., Wang W., Wang S., Zhou W., Ling L. (2024). Multiple dietary patterns and the association between long-term air pollution exposure with type 2 diabetes risk: Findings from UK Biobank cohort study. Ecotoxicol. Environ. Saf..

[36] Ma J., Yao Y., Xie Y., Yin H., Yang S., Shang B. (2025). Omega-3 modify the adverse effects of long-term exposure to ambient air pollution on the incidence of chronic obstructive pulmonary disease: evidence from a nationwide prospective cohort study. Environ. Health..

[37] Page M.J., McKenzie J.E., Bossuyt P.M., Boutron I., Hoffmann T.C., Mulrow C.D. (2021). The PRISMA 2020 statement: an updated guideline for reporting systematic reviews. BMJ.

[38] Page M.J., Moher D., Bossuyt P.M., Boutron I., Hoffmann T.C., Mulrow C.D. (2021). PRISMA 2020 explanation and elaboration: updated guidance and exemplars for reporting systematic reviews. BMJ.

[39] Sheikhalishahi S., Miotto R., Dudley J.T., Lavelli A., Rinaldi F., Osmani V. (2019). Natural language processing of clinical notes on chronic diseases: systematic review. JMIR Med. Inform..

[40] Carbone A. (2020). Cancer classification at the crossroads. Cancers.

[41] Xie X., Li X., Tang W., Xie P., Tan X. (2021). Primary tumor location in lung cancer: the evaluation and administration. Chin. Med. J..

[42] Wu Q., Li G., Zhao C.Y., Na X.L., Zhang Y.B. (2023). Association between phthalate exposure and obesity risk: a meta-analysis of observational studies. Environ. Toxicol. Pharmacol..

[43] Zhu Y., Wang N.N., Pan D., Wang S. (2024). Risk of overweight and obesity in children and adolescents with attention-deficit/hyperactivity disorder: a systematic review and meta-analysis. Childhood Obes.

[44] Alavi M., Hunt G.E., Visentin D.C., Watson R., Thapa D.K., Cleary M. (2020). Using risk and odds ratios to assess effect size for meta-analysis outcome measures. J. Adv. Nurs..

[45] Chinn S. (2000). A simple method for converting an odds ratio to effect size for use in meta-analysis. Stat. Med..

[46] Berlin J.A., Longnecker M.P., Greenland S. (1993). Meta-analysis of epidemiologic dose-response data. Epidemiology.

[47] Higgins J.P.T., Thomas J., Chandler J., Cumpston M., Li T., Page M.J. (2024).

[48] Fisher D.J., Carpenter J.R., Morris T.P., Freeman S.C., Tierney J.F. (2017). Meta-analytical methods to identify who benefits most from treatments: daft, deluded, or deft approach?. BMJ.

[49] Mishra S., Vaartjes I., van der Schouw Y.T., Bijnens E.E.M., Boer J.M.A., Downward G.S. (2025). Air pollution exposure and incidence of cardiometabolic diseases: exploring the modifying role of dietary antioxidant intake in adults. Health. Place..

[50] Cheng Y.J., Zhu C., Deng H., Wu Y., Wei H.Q., Lin W.D. (2025). Air pollution, genetic susceptibility, and the risk of ventricular arrhythmias: a prospective cohort study in the UK Biobank. Eur. J. Prev. Cardiol..

[51] Ji W., Li L., Cheng Y., Yuan Y., Zhao Y., Wang K. (2025). Air pollution, lifestyle, and cardiovascular disease risk in northwestern China: a cohort study of over 5.8 million participants. Environ. Int..

[52] Zhang J., Gu T., Jin J., Han W., Kong Q., Huang J. (2025). Air pollution, MIND diet, risk of neurodegenerative diseases in different aging status. Environ. Health. (Wash)..

[53] Zhang J., Cai L., Gui Z., Wang S., Zeng X., Lai L. (2020). Air pollution-associated blood pressure may be modified by diet among children in Guangzhou, China. J. Hypertens..

[54] Yang Y., Guo Y., Qian Z.M., Ruan Z., Zheng Y., Woodward A. (2018). Ambient fine particulate pollution associated with diabetes mellitus among the elderly aged 50 years and older in China. Environ. Pollut..

[55] Xu Y., Chen J.T., Holland I., Yanosky J.D., Liao D., Coull B.A. (2021). Analysis of long- and medium-term particulate matter exposures and stroke in the US-based Health Professionals Follow-up Study. Environ. Epidemiol..

[56] Guo Q., Xue T., Jia C., Wang B., Cao S., Zhao X. (2020). Association between exposure to fine particulate matter and obesity in children: a national representative cross-sectional study in China. Environ. Int..

[57] Shin M.K., Kim K.N. (2023). Association between long-term air pollution exposure and development of diabetes among community-dwelling adults: modification of the associations by dietary nutrients. Environ. Int..

[58] Liu X., Tu R., Qiao D., Niu M., Li R., Mao Z. (2020). Association between long-term exposure to ambient air pollution and obesity in a Chinese rural population: the Henan Rural Cohort Study. Environ. Pollut..

[59] Guo C.G., Liu Y., Zhang F. (2025). Association of air pollution with ischemic heart disease, stroke, diabetes, COPD, lung cancer, and all-cause mortality: effect modification by pro-inflammatory diet. Glob Transitions.

[60] Wang Y., Liu F., Yao Y., Chen M., Wu C., Yan Y. (2022). Associations of long-term exposure to ambient air pollutants with metabolic syndrome: the Wuhan Chronic Disease Cohort Study. Environ. Res..

[61] Li N., Chen G., Liu F., Mao S., Liu Y., Hou Y. (2019). Associations of long-term exposure to ambient PM_1_ with hypertension and blood pressure in rural Chinese population: the Henan rural cohort study. Environ. Int..

[62] Chen C., Whitsel E.A., Espeland M.A., Snetselaar L., Hayden K.M., Lamichhane A.P. (2022). B vitamin intakes modify the association between particulate air pollutants and incidence of all-cause dementia: findings from the Women’s Health Initiative Memory Study. Alzheimers Dement.

[63] Andersen Z.J., Hvidberg M., Jensen S.S., Ketzel M., Loft S., Sørensen M. (2011). Chronic obstructive pulmonary disease and long-term exposure to traffic-related air pollution: a cohort study. Am. J. Respir. Crit. Care. Med..

[64] Li X., Zhang W., Laden F., Curhan G.C., Rimm E.B., Guo X. (2022). Dietary nitrate intake and vegetable consumption, ambient particulate matter, and risk of hypertension in the Nurses’ Health Study. Environ. Int..

[65] Xu H., Guo B., Qian W., Ciren Z., Guo W., Zeng Q. (2021). Dietary pattern and long-term effects of particulate matter on blood pressure: a large cross-sectional study in Chinese adults. Hypertension.

[66] Chen T., Song L., Zhong X., Zhu Q., Huo J., Chen J. (2023). Dietary polyunsaturated fatty acids intake, air pollution, and the risk of lung cancer: a prospective study in UK Biobank. Sci. Total Environ..

[67] Zhu G., Wen Y., Liang J., Wang T. (2024). Effect modification of diet and vitamins on the association between air pollution particles of different diameters and hypertension: a 12-year longitudinal cohort study in densely populated areas of China. Sci. Total Environ..

[68] Zheng G., Xia H., Shi H., Zheng D., Wang X., Ai B. (2024). Effect modification of dietary diversity on the association of air pollution with incidence, complications, and mortality of type 2 diabetes: results from a large prospective cohort study. Sci. Total Environ..

[69] Zheng X.Y., Tang S.L., Liu T., Wang Y., Xu X.J., Xiao N. (2022). Effects of long-term PM_2.5_ exposure on metabolic syndrome among adults and elderly in Guangdong, China. Environ. Health..

[70] Guo B., Guo Y., Nima Q., Feng Y., Wang Z., Lu R. (2022). Exposure to air pollution is associated with an increased risk of metabolic dysfunction-associated fatty liver disease. J. Hepatol..

[71] Li D., Xie J., Wang L., Sun Y., Hu Y., Tian Y. (2023). Genetic susceptibility and lifestyle modify the association of long-term air pollution exposure on major depressive disorder: a prospective study in UK Biobank. BMC Med.

[72] Zhu S., Zhang X., Wu Z., Jin Y., Wu W., Zhang J. (2025). Healthful plant-based dietary patterns, PM_2.5_ exposure and the risk of heart failure: a population-based cohort study. Br. J. Nutr..

[73] Cai H., Du Z., Lin X., Lawrence W.R., Hopke P.K., Rich D.Q. (2023). Interactions between long-term ambient particle exposures and lifestyle on the prevalence of hypertension and diabetes: insight from a large community-based survey. J. Epidemiol. Community Health..

[74] Li H., Cai M., Li H., Qian Z.M., Stamatakis K., McMillin S.E. (2022). Is dietary intake of antioxidant vitamins associated with reduced adverse effects of air pollution on diabetes? Findings from a large cohort study. Ecotoxicol. Environ. Saf..

[75] Mao S., Li S., Wang C., Liu Y., Li N., Liu F. (2020). Is long-term PM_1_ exposure associated with blood lipids and dyslipidemias in a Chinese rural population?. Environ. Int..

[76] Jin J., Xu Z., Beevers S.D., Huang J., Kelly F., Li G. (2024). Long-term ambient ozone, omega-3 fatty acid, genetic susceptibility, and risk of mental disorders among middle-aged and older adults in UK Biobank. Environ. Res..

[77] Lin H., Guo Y., Zheng Y., Di Q., Liu T., Xiao J. (2017). Long-term effects of ambient PM_2.5_ on hypertension and blood pressure and attributable risk among older Chinese adults. Hypertension.

[78] Li F.R., Wu K.Y., Fan W.D., Chen G.C., Tian H., Wu X.B. (2022). Long-term exposure to air pollution and risk of incident inflammatory bowel disease among middle and old aged adults. Ecotoxicol. Environ. Saf..

[79] Yang B.Y., Qian Z.M., Li S., Fan S., Chen G., Syberg K.M. (2018). Long-term exposure to ambient air pollution (including PM_1_) and metabolic syndrome: the 33 Communities Chinese Health Study (33CCHS). Environ. Res..

[80] Zhang Z., Laden F., Forman J.P., Hart J.E. (2016). Long-term exposure to particulate matter and self-reported hypertension: a prospective analysis in the Nurses’ Health Study. Environ. Health Perspect..

[81] Raaschou-Nielsen O., Andersen Z.J., Hvidberg M., Jensen S.S., Ketzel M., Sørensen M. (2011). Lung cancer incidence and long-term exposure to air pollution from traffic. Environ. Health Perspect..

[82] Lamichhane D.K., Kim H.C., Choi C.M., Shin M.H., Shim Y.M., Leem J.H. (2017). Lung cancer risk and residential exposure to air pollution: a Korean population-based case-control study. Yonsei Med. J..

[83] Margetaki K., Bempi V., Michalaki E., Roumeliotaki T., Iakovides M., Stephanou E. (2024). Prenatal air pollution exposure and childhood obesity: effect modification by maternal fruits and vegetables intake. Int. J. Hyg. Environ. Health..

[84] Zhang C., Wang Y., Xie W., Zhang J., Tian T., Zhu Q. (2024). Sex differences and dietary patterns in the association of air pollutants and hypertension. BMC Public Health.

[85] Kwak J.H., Kim H.J. (2023). The association between air pollutants exposure with pre- and hypertension by vitamin C intakes in Korean adults: a cross-sectional study from the 2013-2016 Korea National Health and Nutrition Examination. J. Nutr. Health. Aging..

[86] Chen Y., Guo C., Chung M.K., Yi Q., Wang X., Wang Y. (2024). The associations of prenatal exposure to fine particulate matter and its chemical components with allergic rhinitis in children and the modification effect of polyunsaturated fatty acids: a birth cohort study. Environ. Health. Perspect..

[87] Lei J.X., Li S., Li X.X., Yin L.H., Zhang F.Y., Zhan Q.Q. The protective effect of the composite dietary antioxidant index on hypertension risk associated with long-term exposure to PM_2.5_ and its constituents: a 10-year cohort study in southwest China[preprint].

[88] Li J.M., Yang H.Y., Wu S.H., Dharmage S.C., Jalaludin B., Knibbs L.D. (2023). The associations of particulate matter short-term exposure and serum lipids are modified by vitamin D status: a panel study of young healthy adults. Environ. Pollut..

[89] Zu P., Zhou L., Yin W., Zhang L., Wang H., Xu J. (2023). Association between exposure to air pollution during preconception and risk of gestational diabetes mellitus: the role of anti-inflammatory diet. Environ. Res..

[90] Cao Y., Liu Y., Ma M., Cai J., Liu M., Zhang R. (2024). Moderating effect of a sodium-rich diet on the association between long-term exposure to fine particulate matter and blood lipids in children and adolescents. BMC Pediatr.

[91] Zhu A., Chen H., Shen J., Wang X., Li Z., Zhao A. (2022). Interaction between plant-based dietary pattern and air pollution on cognitive function: a prospective cohort analysis of Chinese older adults. Lancet Reg. Health West Pac..

[92] Zu P., Zhang L., Zhang K., He L., Fan Y., Zhou C. (2024). Anti-inflammatory diet mitigate cardiovascular risks due to particulate matter exposure in women during pregnancy: a perspective cohort study from China. Environ. Res..

[93] Hehua Z., Yang X., Qing C., Shanyan G., Yuhong Z. (2021). Dietary patterns and associations between air pollution and gestational diabetes mellitus. Environ. Int..

[94] Barthelemy J., Sanchez K., Miller M.R., Khreis H. (2020). New opportunities to mitigate the burden of disease caused by traffic related air pollution: antioxidant-rich diets and supplements. Int. J. Environ. Res. Public Health..

[95] Aune D., Metoudi M., Sadler I., Kassam S. (2025). Whole grain and refined grain consumption and the risk of hypertension: a systematic review and meta-analysis of prospective studies. Sci. Rep..

[96] Mellen P.B., Walsh T.F., Herrington D.M. (2008). Whole grain intake and cardiovascular disease: a meta-analysis. Nutr. Metab. Cardiovasc. Dis..

[97] Feng Y., Zhao Y., Liu J., Huang Z., Yang X., Qin P. (2022). Consumption of dairy products and the risk of overweight or obesity, hypertension, and type 2 diabetes mellitus: a dose-response meta-analysis and systematic review of cohort studies. Adv. Nutr..

[98] Chen Z., Ahmed M., Ha V., Jefferson K., Malik V., Ribeiro P.A.B. (2022). Dairy product consumption and cardiovascular health: a systematic review and meta-analysis of prospective cohort studies. Adv. Nutr..

[99] Xi B., Huang Y., Reilly K.H., Li S., Zheng R., Barrio-Lopez M.T. (2015). Sugar-sweetened beverages and risk of hypertension and CVD: a dose-response meta-analysis. Br. J. Nutr..

[100] Zhao Y., Feng Y., Zeng Y., Di W., Luo X., Wu X. (2024). Sugar intake and risk of hypertension: a systematic review and dose-response meta-analysis of cohort and cross-sectional studies. Crit. Rev. Food. Sci. Nutr..

[101] Farhangi M.A., Nikniaz L., Khodarahmi M. (2020). Sugar-sweetened beverages increases the risk of hypertension among children and adolescence: a systematic review and dose-response meta-analysis. J. Transl. Med..

[102] Rocha-Velasco O.A.S., Morales-Suárez-Varela M.A., lopis-González A.N. (2024). Dietary flavonoids: mitigating air pollution's cardiovascular risks. Nutrients.

[103] Torres-Gonzalez M., Rice Bradley B.H. (2023). Whole-milk dairy foods: biological mechanisms underlying beneficial effects on risk markers for cardiometabolic health. Adv. Nutr..

[104] Jia G., Aroor A.R., Whaley-Connell A.T., Sowers J.R. (2014). Fructose and uric acid: is there a role in endothelial function?. Curr. Hypertens. Rep..

[105] Mensink-Bout S.M., van Meel E.R., de Jongste J.C., Annesi-Maesano I., Aubert A.M., Bernard J.Y. (2022). Maternal diet in pregnancy and child's respiratory outcomes: an individual participant data meta-analysis of 18 000 children. Eur. Respir. J..

[106] Fowler M.E., Akinyemiju T.F. (2017). Meta-analysis of the association between dietary inflammatory index (DII) and cancer outcomes. Int. J. Cancer..

[107] Péter S., Holguin F., Wood L.G., Clougherty J.E., Raederstorff D., Antal M. (2015). Nutritional solutions to reduce risks of negative health impacts of air pollution. Nutrients.

[108] Kim Y., Kim J. (2020). N-6 polyunsaturated fatty acids and risk of cancer: accumulating evidence from prospective studies. Nutrients.

[109] Anandan C., Nurmatov U., Sheikh A. (2009). Omega 3 and 6 oils for primary prevention of allergic disease: systematic review and meta-analysis. Allergy.

[110] Ye E., Xu Z., Hou X., Wang Y., Zhou C., Xiang J. (2025). Joint effects of air pollution and diet patterns on the risk of chronic obstructive pulmonary disease. Sci. Rep..

[111] Lemoine S C.M., Brigham E.P., Woo H., Hanson C.K., McCormack M.C., Koch A. (2019). Omega-3 fatty acid intake and prevalent respiratory symptoms among U.S. adults with COPD. BMC Pulm. Med..

[112] Li J., Chen Y., Shi Q., Sun J., Zhang C., Liu L. (2023). Omega-3 polyunsaturated fatty acids ameliorate PM_2.5_ exposure induced lung injury in mice through remodeling the gut microbiota and modulating the lung metabolism. Environ. Sci. Pollut. Res..

[113] Tong H., Zhang S., Shen W., Chen H., Salazar C., Schneider A. (2022). Lung function and short-term ambient air pollution exposure: differential impacts of omega-3 and omega-6 fatty acids. Ann. Am. Thorac. Soc..

[114] Miles E.A., Calder P.C. (2014). Omega-6 and omega-3 polyunsaturated fatty acids and allergic diseases in infancy and childhood. Curr. Pharm. Des..

[115] Larruy-García A., Mahmood L., Miguel-Berges M.A.L., Masip G., Seral-Cortés M., De Miguel-Etayo P. (2024). Diet quality scores, obesity and metabolic syndrome in children and adolescents: a systematic review and meta-analysis. Curr. Obes. Rep..

[116] Jayedi A., Soltani S., Abdolshahi A., Shab-Bidar S. (2020). Healthy and unhealthy dietary patterns and the risk of chronic disease: an umbrella review of meta-analyses of prospective cohort studies. Br. J. Nutr..

[117] Malik V.S., Popkin B.M., Bray G.A., Després J.P., Willett W.C., Hu F.B. (2010). Sugar-sweetened beverages and risk of metabolic syndrome and type 2 diabetes: a meta-analysis. Diabetes Care.

[118] Zhang X., Li X., Liu L., Hong F., Zhao H., Chen L. (2021). Dose-response association between sugar- and artificially sweetened beverage consumption and the risk of metabolic syndrome: a meta-analysis of population-based epidemiological studies. Public Health Nutr.

[119] Zheng G., Chen L., Ran S., Wei S., Ho K.F., Huang Z. (2025). A hypothetical intervention analysis for the effects of healthy dietary patterns on reducing major chronic diseases and mortality associated with air pollutant mixtures. BMC Med.

[120] Qin Y., Zhang H., Jiang B., Chen J., Zhang T. (2023). Food bioactives lowering risks of chronic diseases induced by fine particulate air pollution: a comprehensive review. Crit. Rev. Food Sci. Nutr..

[121] Tsompanaki E., Thanapirom K., Papatheodoridi M., Parikh P., Chotai De Lima Y., Tsochatzis E.A. (2023). Systematic review and meta-analysis: the role of diet in the development of nonalcoholic fatty liver disease. Clin. Gastroenterol. Hepatol..

[122] Tarantino G., Capone D., Finelli C. (2013). Exposure to ambient air particulate matter and non-alcoholic fatty liver disease. World J. Gastroenterol..

