# Supplementary material for: Dietary Intake Modifies the Association between Air Pollution and Chronic Diseases: A Systematic Review and Meta-analysis
Source: Adv Nutr. 2026 Jul 20;17(9):100709. doi: 10.1016/j.advnut.2026.100709 (PMC13486347; doi:10.1016/j.advnut.2026.100709)
Supplement: Supplementary file 1 [file mmc1.docx]

**Title:** Dietary Intake Modifies the Association Between Air Pollution and Chronic Diseases: A Systematic Review and Meta-Analysis

**First Author:** Ye Chen

**Supplemental Material**

**Dietary Intake Modifies the Association Between Air Pollution and Chronic Diseases: A Systematic Review and Meta-Analysis**

Ye Chen, Bangqin Cai, Fei Wang, Hongyu Xuan, Yongqiang Sun, Yang Xia, Yuhong Zhao, Lishen Shan*, Hehua Zhang*

**Table of contents**

**Supplementary Figure 1.** Sensitivity analysis of the effect modification by dietary intake on the association between air pollution and chronic disease risk in children.

**Supplementary Figure 2.** Sensitivity analysis of the effect modification by dietary intake on the association between air pollution and chronic disease risk in adults.

**Supplementary Table 1.** Detailed search strategies in different databases.

**Supplementary Table 2.** The classification of diseases included in the study.

**Supplementary Table 3.** Characteristics of included studies (N = 51).

**Supplementary Table 4.** Dietary factors corresponding to diseases of various systems.

**Supplementary Table 5.** Quality assessments of included cohort studies (N = 30).

**Supplementary Table 6.** Quality assessments of included case-control studies (N = 1).

**Supplementary Table 7.** Quality assessments of included cross-sectional studies (N = 20).

**Supplementary Table 8.** The modifying effect of the dietary item in a single study.

**Supplementary Figure 1.** Sensitivity analysis of the effect modification by dietary intake on the association between air pollution and chronic disease risk in children.

Note: * Deft analysis represents the pooled within-study interaction effect, expressed as the meta-analyzed log ratio of odds ratios with corresponding 95% confidence intervals. Abbreviations: CI: confidence interval; CRD: chronic respiratory diseases; CVD: cardiovascular diseases; FV: fruits and vegetables; MD: metabolic diseases; N: number of data entries in meta-analysis; PM: particulate matter; pOR: pooled odds ratios; *P*_int_, *P* value for interaction; SSB: sugar-sweetened beverage.

**Supplementary Figure 2.** Sensitivity analysis of the effect modification by dietary intake on the association between air pollution and chronic disease risk in adults.

Note: * Deft analysis represents the pooled within-study interaction effect, expressed as the meta-analyzed log ratio of odds ratios with corresponding 95% confidence intervals. Abbreviations: CI: confidence interval; CRD: chronic respiratory diseases; CVD: cardiovascular diseases; FV: fruits and vegetables; MD: metabolic diseases; N: number of data entries in meta-analysis; PM: particulate matter; pOR: pooled odds ratios; *P*_int_, *P* value for interaction; SSB: sugar-sweetened beverage.

**Supplementary Table 1.** Detailed search strategies in different databases.

| **PubMed** | |
| --- | --- |
| #1 | (diet*) OR (nutrition) OR (food) OR (nutrient) OR (nutriment) OR (nutritive) OR (macronutrient*) OR (fat) OR (protein) OR (carbohydrate) OR (dietary fiber) OR (fiber) OR (micronutrient*) OR (vitamin*) OR (supplement*) OR (‘dietary pattern’) |
| #2 | (‘air pollution’) OR (‘traffic-related pollution’) OR (‘particulate matter’) OR (‘air quality’) OR (‘traffic pollution’) OR (‘vehicle pollution’) OR (‘airborne particulate matter’) OR (‘ambient particulate matter’) OR (‘air pollutants’) OR (‘particulate air pollutants’) OR (‘ultrafine particulate matter’) OR (‘ultrafine particle’) |
| #3 | (interaction) OR (modify) OR (modification) OR (Mediat*) OR (interplay) OR (intermedi*) |
| #4 | #1 AND #2 AND #3 |
| **Embase** | |
| #1 | ‘diet’/exp OR ‘nutrition’/exp OR ‘food’/exp OR ‘nutrient’: ab,ti,kw OR ‘nutrimen’: ab,ti,kw OR ‘nutritive’: ab,ti,kw OR ‘macronutrient*’: ab,ti,kw OR ‘fat’: ab,ti,kw OR ‘protein’: ab,ti,kw OR ‘carbohydrate’: ab,ti,kw OR ‘dietary fiber’: ab,ti,kw OR ‘fiber’: ab,ti,kw OR ‘micronutrient*’: ab,ti,kw OR ‘vitamin*’: ab,ti,kw OR ‘supplement*’: ab,ti,kw OR ‘dietary pattern’: ab,ti,kw OR 'diet regimen': ab,ti,kw OR 'diet surveys': ab,ti,kw OR 'dietary regimen': ab,ti,kw OR 'dietary survey': ab,ti,kw OR 'nutrition survey': ab,ti,kw |
| #2 | ‘air pollution’/exp OR ‘traffic pollution’/exp OR ‘particulate matter’: ab,ti,kw OR ‘aerial pollution’: ab,ti,kw OR ‘aerogenic pollution’: ab,ti,kw OR ‘air contamination’: ab,ti,kw OR ‘airborne pollution’: ab,ti,kw OR ‘atmosphere pollution’: ab,ti,kw OR ‘atmospheric pollution’: ab,ti,kw OR ‘traffic-related pollution’: ab,ti,kw OR ‘vehicle-related pollution’: ab,ti,kw OR ‘air-borne pollution’: ab,ti,kw |
| #3 | ‘interaction’: ab,ti,kw OR ‘modify’: ab,ti,kw OR ‘modification’: ab,ti,kw OR ‘Mediat*’: ab,ti,kw OR ‘interplay’: ab,ti,kw OR ‘intermedi*’: ab,ti,kw |
| #4 | #1 AND #2 AND #3 |
| **Web of Science** | |
| #1 | TS= ((diet*) OR (nutrition) OR (food) OR (nutrient) OR (nutriment) OR (nutritive) OR (macronutrient*) OR (fat) OR (protein) OR (carbohydrate) OR (dietary fiber) OR (fiber) OR (vitamin*) OR (micronutrient*) OR (supplement*) OR (‘dietary pattern’)) |
| #2 | TS= ((air pollution) OR (‘traffic-related pollution’) OR (‘particulate matter’) OR (‘air quality’) OR (‘traffic pollution’) OR (‘vehicle pollution’) OR (‘airborne particulate matter’) OR (‘ambient particulate matter’) OR (‘air pollutants’) OR (‘particulate air pollutants’) OR (‘ultrafine particulate matter’) OR (‘ultrafine particle’)) |
| #3 | TS= ((interaction) OR (modify) OR (modification) OR (Mediat*) OR (interplay) OR (intermedi*)) |
| #4 | #1 AND #2 AND #3 |
| **Cochrane** | |
| #1 | (diet*): ab,ti,kw OR (nutrition): ab,ti,kw OR (food): ab,ti,kw OR (nutrient): ab,ti,kw OR (nutrimen): ab,ti,kw OR (nutritive): ab,ti,kw OR (macronutrient*): ab,ti,kw OR (fat): ab,ti,kw OR (protein): ab,ti,kw OR (carbohydrate): ab,ti,kw OR (dietary fiber): ab,ti,kw OR (fiber): ab,ti,kw OR (micronutrient*): ab,ti,kw OR (vitamin*): ab,ti,kw OR (supplement*): ab,ti,kw OR (dietary pattern): ab,ti,kw |
| #2 | (air pollution): ab,ti,kw OR (traffic-related pollution): ab,ti,kw OR (vehicle emissions): ab,ti,kw OR (traffic-related pollutants): ab,ti,kw OR (transportation emissions): ab,ti,kw OR (particulate matter): ab,ti,kw OR (air quality): ab,ti,kw OR (traffic pollution): ab,ti,kw OR (vehicle pollution): ab,ti,kw OR (airborne particulate matter): ab,ti,kw OR (ambient particulate matter): ab,ti,kw OR (air pollutants): ab,ti,kw OR (particulate air pollutants): ab,ti,kw OR (ultrafine particulate matter): ab,ti,kw OR (ultrafine particle): ab,ti,kw |
| #3 | (interaction): ti,ab,kw OR (modify): ti,ab,kw OR (modification): ti,ab,kw OR (Mediat*): ti,ab,kw OR (interplay): ti,ab,kw OR (intermedi*): ti,ab,kw |
| #4 | #1 AND #2 AND #3 |

**Supplementary Table 2.** The classification of diseases included in the study.

| **Disease systems** | **ICD-10 code** | **Specific diseases** |
| --- | --- | --- |
| Cardiovascular diseases | I00-I99 | Heart failure; Hypertension; Ventricular arrhythmias; Stroke; Ischemic heart disease; |
| Chronic respiratory diseases | J30-J99; C34 | Chronic obstructive pulmonary disease; Allergic rhinitis; Lung cancer; |
| Metabolic disorders | E00-E90 | Diabetes; Metabolic syndrome; Obesity; Hypertriglyceridemia; Gestational diabetes mellitus; Hypoalphalipo-proteinemia; Hyperbetalipo-proteinemia; Dyslipidemia; |
| Neuropsychiatric disorders | F00-F99; G00-G99 | Neurodegenerative diseases; Cognitive dysfunction; Depression; Dementia; |
| Digestive diseases | K00-K93 | Crohn’s disease; Ulcerative colitis; Metabolic dysfunction-associated fatty liver disease; |

**Supplementary Table 3.** Characteristics of included studies (N = 51).

| **Author, year** | **Country** | **Follow-up time** | **Age** | **Study design** | **Samples** | **Dietary intake** | **Pollutants** | **Outcome** | **Effect size** | **Covariates adjusted** |
| --- | --- | --- | --- | --- | --- | --- | --- | --- | --- | --- |
| Mishra et al. (2025) | Dutch | 17 years | 50.1 (11.3) y | Cohort | 30,519 | Vitamin C (VC); Vitamin E (VE) | PM_2.5_; PM_10_; | Heart failure (HF) | PM_2.5_:  Total HR (95%CI): 0.38 (0.11, 1.26);  High VC: 1.18 (0.26, 5.38);  Low VC: 0.09 (0.01, 0.59);  PM_10_:  Total HR (95%CI): 1.97 (0.83, 4.70);  High VE: 0.69 (0.17, 2.74);  Low VE: 4.24 (1.37, 13.15); | Age, sex, educational level, smoking status, alcohol consumption, physical activity, total dietary fiber intake, total dietary energy intake, Body Mass Index, and area-level socioeconomic information. |
| Huang et al. (2023) | China | — | ≥ 18 y | Cross-sectional | 47,501 | DASH score; MED; Fruits; Vegetables; Dairy; Sodium; Legumes and nuts; Red meat; Whole grain; Fish | PM_2.5_ | Hypertension | PM_2.5_:  Total OR (95%CI): 1.08 (1.03, 1.12);  High DASH score: 0.98 (0.91, 1.05);  Low DASH score: 1.20 (1.10, 1.30);  Pint < 0.01;  High MED: 1.04 (0.97, 1.11);  Low MED: 1.19 (1.09, 1.29);  Pint = 0.030;  High fruits: 1.02 (0.95, 1.09);  Low fruits: 1.14 (1.05, 1.23);  Pint < 0.01;  High vegetables: 1.05 (0.98, 1.13);  Low vegetables: 1.13 (1.05, 1.21);  Pint = 0.030;  High dairy: 1.00 (0.93, 1.07);  Low dairy: 1.12 (1.07, 1.18);  Pint < 0.01;  High sodium: 1.12 (1.04, 1.20);  Low sodium: 1.08 (1.00, 1.16);  Pint = 0.570;  High legumes and nuts: 1.09 (1.01, 1.17);  Low legumes and nuts: 1.15 (1.06, 1.24);  Pint = 0.260;  High red meat: 1.13 (1.05, 1.22);  Low red meat: 1.11 (1.03, 1.20);  Pint = 0.260;  High whole grain: 1.00 (0.94, 1.07);  Low whole grain: 1.18 (1.11, 1.25);  Pint < 0.01;  High fish: 1.16 (1.07, 1.26);  Low fish: 1.04 (0.97, 1.12);  Pint = 0.280; | Age, sex, education, marital status, annual household income, smoking status, passive smoking, alcohol status, physical activity, outdoor time, body mass index, household solid fuel use, HTN family history, residence, and region. |
| Kwak et al. (2023) | Korea | — | ≥ 20 y | Cross-sectional | 11,866 | VC | PM_10_; | Hypertension | PM_10_:  Total OR (95%CI): 1.27 (1.13, 1.44);  High VC: 1.16 (1.03, 1.30);  Low VC: 1.23 (1.08, 1.40); | Age, sex, education, exercise status, alcohol consumption, smoking status, family income, BMI, and survey-years |
| Li (a) et al. (2022) | USA | 766,834 person-years | 30-55 y | Cohort | 55,232 | Green leafy vegetables servings (GLV) | PM_2.5_; PM_10_ | Hypertension | PM_2.5_:  Total HR (95%CI): 1.06 (1.02, 1.11);  High GLV: 1.14 (1.06, 1.24);  Low GLV: 1.00 (0.92, 1.08);  Pint = 0.004;  PM_10_:  Total HR (95%CI): 1.01 (0.99, 1.03);  High GLV: 1.03 (0.99, 1.07);  Low GLV: 1.02 (0.98, 1.07);  Pint = 0.140; | Age, race, biennial follow-up interval, family history of hypertension, body mass index, smoking status, physical activity, neighborhood socioeconomic status score, marital status, Registered Nursing degree, husband’s educational attainment, menopausal status and postmenopausal hormone use, total energy consumption (quartiles), Dietary Approaches to Stop Hypertension diet score, alcohol intake, folate intake and non-narcotic analgesic use. |
| Xu (a) et al. (2021) | China | — | 50.65 y | Cross-sectional | 61,081 | DASH score; | PM_1_; PM_2.5_; PM_10_ | Hypertension | PM_1_:  Total OR (95%CI): 1.11 (1.03, 1.20);  High DASH score: 1.06 (0.95, 1.19);  Low DASH score: 1.20 (1.08, 1.32);  Pint = 0.005;  PM_2.5_:  Total OR (95%CI): 1.10 (1.06, 1.15);  High DASH score: 1.07 (1.02, 1.13);  Low DASH score: 1.15 (1.09, 1.20);  Pint = 0.001;  PM_10_:  Total OR (95%CI): 1.05 (1.02, 1.08);  High DASH score: 1.04 (1.00, 1.08);  Low DASH score: 1.08 (1.05, 1.12);  Pint = 0.001; | Age, sex, annual family income, education level, marital status, region, ethnic group, alcohol status, smoking status, secondary smoking, indoor air pollution, physical activity, DASH score, body mass index, hypertension family history and 2 meteorologic characteristics. |
| Zhang et al. (2020) | China | — | 9.1 (1.7) y | Cross-sectional | 7,225 | Vegetables; Fruits; Meat; Fish; Sugar-sweetened beverages (SSB); Milk; Grains; Soybean products | PM_2.5_; PM_10_; SO_2_; O_3_ | Hypertension | PM_2.5_:  Total OR (95%CI): 1.33 (1.11, 1.61);  High vegetables: 1.37 (1.07, 1.75);  Low vegetables: 1.31 (1.03, 1.66);  Pint = 0.418;  High fruits: 1.29 (1.02, 1.64);  Low fruits: 1.41 (1.08, 1.84);  Pint = 0.688;  High meat: 1.42 (1.08, 1.86);  Low meat: 1.25 (1.00, 1.57);  Pint = 0.245;  High fish: 1.38 (1.07, 1.78);  Low fish: 1.29 (1.01, 1.63);  Pint = 0.561;  High SSB: 1.34 (1.05, 1.71);  Low SSB: 1.35 (1.05, 1.73);  Pint = 0.786;  High milk: 1.32 (1.04, 1.68);  Low milk: 1.39 (1.06, 1.82)  Pint: 0.427;  High grains: 1.27 (1.00, 1.62);  Low grains: 1.39 (1.08, 1.80);  Pint = 0.585;  High soybean products: 1.46 (1.12, 1.90);  Low soybean products: 1.25 (0.99, 1.59);  Pint = 0.215;  PM_10_:  Total OR (95%CI): 1.01 (0.75, 1.35);  High vegetables: 0.87 (0.57, 1.33);  Low vegetables: 1.13 (0.71, 1.80);  Pint = 0.710;  High fruits: 0.90 (0.59, 1.36);  Low fruits: 1.25 (0.76, 2.06);  Pint = 0.722;  High meat: 1.12 (0.74, 1.68);  Low meat: 0.90 (0.63, 1.28);  Pint = 0.127;  High fish: 0.90 (0.58, 1.39);  Low fish: 1.15 (0.74, 1.81);  Pint = 0.988;  High SSB: 1.11 (0.71, 1.74);  Low SSB: 0.98 (0.63, 1.53);  Pint = 0.600;  High milk: 1.08 (0.71, 1.66);  Low milk: 0.97 (0.60, 1.55);  Pint = 0.969;  High grains: 1.10 (0.75, 1.59);  Low grains: 0.94 (0.64, 1.39);  Pint = 0.375;  High soybean products: 1.06 (0.71, 1.60);  Low soybean products: 1.00 (0.69, 1.43);  Pint = 0.581;  SO_2_:  Total OR (95%CI): 1.70 (1.33, 2.16);  High vegetables: 1.12 (0.77, 1.62);  Low vegetables: 1.34 (0.91, 1.95);  Pint = 0.772;  High fruits: 1.20 (0.85, 1.71);  Low fruits: 1.18 (0.79, 1.75);  Pint = 0.344;  High meat: 1.81 (1.14, 2.89);  Low meat: 1.61 (1.24, 2.08);  Pint = 0.435;  High fish: 1.30 (0.89, 1.90);  Low fish: 1.19 (0.83, 1.70);  Pint = 0.756;  High SSB: 1.06 (0.74, 1.51);  Low SSB: 1.35 (0.92, 1.98);  Pint = 0.315;  High milk: 1.57 (1.17, 2.10);  Low milk: 1.89 (1.31, 2.75);  Pint = 0.349;  High grains: 1.08 (0.75, 1.55);  Low grains: 1.33 (0.90, 1.98);  Pint = 0.343;  High soybean products: 1.34 (0.90, 1.97);  Low soybean products: 1.28 (0.69, 2.37);  Pint = 0.155;  O_3_:  Total OR (95%CI): 1.48 (1.20, 1.83);  High vegetables: 1.13 (0.76, 1.67);  Low vegetables: 1.36 (0.96, 1.91);  Pint = 0.564;  High fruits: 1.15 (0.80, 1.65);  Low fruits: 1.39 (0.97, 2.00);  Pint = 0.161;  High meat: 1.14 (0.74, 1.74);  Low meat: 1.33 (0.97, 1.82);  Pint = 0.916;  High fish: 1.34 (0.95, 1.89);  Low fish: 1.22 (0.84, 1.75);  Pint = 0.787;  High SSB: 1.56 (1.22, 1.99);  Low SSB: 1.41 (1.10, 1.81);  Pint = 0.130;  High milk: 0.96 (0.62, 1.48);  Low milk: 1.64 (1.16, 2.32);  Pint = 0.904;  High grains: 1.27 (0.90, 1.78);  Low grains: 1.22 (0.81, 1.82);  Pint = 0.257;  High soybean products: 1.52 (1.06, 2.17);  Low soybean products: 1.07 (0.74, 1.54);  Pint = 0.051; | Age, sex, weight status, humidity, breast, outdoor physical activity time, parental education, parental smoking status, and family history of hypertension. |
| Zhu et al. (2024) | China | 12 years | 41.78 (9.93) y | Cohort | 3,205 | Fruits; Vegetables; Bean; Egg; Fish; Dairy; Meat; SSB; Sodium;  Fungus food;  Whole grains; VC; Vitamin B (VB)_1_; VB_2_; VB_3_; VE | PM_1_; PM_2.5_; PM_10_ | Hypertension | PM_1_:  Total HR (95%CI): 1.02 (1.01, 1.03);  High fruits: 1.01 (1.00, 1.02);  Low fruits: 1.03 (1.02, 1.04);  High vegetables: 1.02 (1.01, 1.03);  Low vegetables: 1.02 (1.01, 1.03);  High bean: 1.02 (1.01, 1.02);  Low bean: 1.02 (1.02, 1.03);  High egg: 1.01 (1.00 1.02);  Low egg: 1.03 (1.02, 1.04);  High fish: 1.02 (1.01, 1.03);  Low fish: 1.02 (1.01, 1.03);  High dairy: 1.01 (1.00, 1.03);  Low dairy: 1.02 (1.02, 1.03);  High meat: 1.02 (1.01, 1.03);  Low meat: 1.02 (1.01, 1.03);  High SSB: 1.02 (1.02, 1.03);  Low SSB: 1.01 (1.00, 1.02);  High sodium: 1.03 (1.02, 1.03);  Low sodium: 1.01 (1.00, 1.02);  High fungus food: 1.01 (1.00, 1.02);  Low fungus food: 1.02 (1.02, 1.03);  High whole grains: 1.01 (1.01, 1.02);  Low whole grains: 1.02 (1.02, 1.03);  High VC: 1.02 (1.01, 1.03);  Low VC: 1.02 (1.01, 1.03);  High VB_1_: 1.02 (1.01, 1.03);  Low VB_1_: 1.02 (1.01, 1.03);  High VB_2_: 1.02 (1.01, 1.03);  Low VB_2_: 1.02 (1.01, 1.03);  High VB_3_: 1.02 (1.01, 1.03);  Low VB_3_: 1.02 (1.01, 1.03);  High VE: 1.01 (1.01, 1.02);  Low VE: 1.03 (1.02, 1.04);  PM_2.5_:  Total HR (95%CI): 1.01 (1.01, 1.02);  High fruits: 1.01 (1.00, 1.01);  Low fruits: 1.02 (1.01, 1.02);  High vegetables: 1.01 (1.01, 1.02);  Low vegetables: 1.01 (1.01, 1.02);  High bean: 1.01 (1.01, 1.02);  Low bean: 1.01 (1.01, 1.02);  High egg: 1.01 (1.00, 1.01);  Low egg: 1.02 (1.01, 1.02);  High fish: 1.01 (1.01, 1.02);  Low fish: 1.01 (1.01, 1.02);  High dairy: 1.01 (1.00, 1.03);  Low dairy: 1.02 (1.01, 1.03);  High meat: 1.01 (1.01, 1.02);  Low meat: 1.01 (1.01, 1.02);  High SSB: 1.01 (1.01, 1.02);  Low SSB: 1.01 (1.00, 1.01);  High sodium: 1.02 (1.01, 1.02);  Low sodium: 1.01 (1.00, 1.01);  High fungus food: 1.01 (1.00, 1.02);  Low fungus food: 1.01 (1.01, 1.02);  High whole grains: 1.01 (1.00, 1.01);  Low whole grains: 1.01 (1.01, 1.02);  High VC: 1.01 (1.01, 1.02);  Low VC: 1.01 (1.01, 1.02);  High VB_1_: 1.01 (1.01, 1.02);  Low VB_1_: 1.01 (1.00, 1.03);  High VB_2_: 1.01 (1.00, 1.02);  Low VB_2_: 1.01 (1.01, 1.02);  High VB_3_: 1.01 (1.01, 1.02);  Low VB_3_: 1.02 (1.01, 1.03);  High VE: 1.01 (1.00, 1.02);  Low VE: 1.03 (1.01, 1.04);  PM_10_:  Total HR (95%CI): 1.01 (1.01, 1.01);  High fruits: 1.00 (1.00, 1.01);  Low fruits: 1.01 (1.01, 1.01);  High vegetables: 1.01 (1.00, 1.01);  Low vegetables: 1.01 (1.01, 1.01);  High bean: 1.01 (1.00, 1.01);  Low bean: 1.01 (1.01, 1.01);  High egg: 1.00 (1.00, 1.01);  Low egg: 1.01 (1.01, 1.01);  High fish: 1.01 (1.00, 1.01);  Low fish: 1.01 (1.01, 1.01);  High dairy: 1.01 (1.00, 1.01);  Low dairy: 1.01 (1.01, 1.01);  High meat: 1.01 (1.01, 1.01);  Low meat: 1.01 (1.00, 1.01);  High SSB: 1.01 (1.01, 1.01);  Low SSB: 1.00 (1.00, 1.01);  High sodium: 1.01 (1.01, 1.01);  Low sodium: 1.00 (1.00, 1.01);  High fungus: 1.01 (1.00, 1.01);  Low fungus: 1.01 (1.01, 1.01);  High whole grain: 1.01 (1.00, 1.01);  Low whole grain: 1.01 (1.00, 1.01);  High VC: 1.01 (1.01, 1.01);  Low VC: 1.01 (1.01, 1.01);  High VB_1_: 1.01 (1.00, 1.01);  Low VB_1_: 1.01 (1.00, 1.01);  High VB_2_: 1.01 (1.00, 1.01);  Low VB_2_: 1.01 (1.00, 1.01);  High VB_3_: 1.01 (1.00, 1.01);  Low VB_3_: 1.01 (1.00, 1.01);  High VE: 1.00 (1.00, 1.01);  Low VE: 1.01 (1.01, 1.01); | Age, household annual income, gender, education level, marital status, smoking status, alcohol status, physical activity, body mass index, residence, sleep duration, and sedentary time. |
| Cai et al. (2023) | China | — | > 45 y | Cross-sectional | 82,345 | Fruits and vegetables (FV); Meat | PM_2.5_; PM_10_ | Hypertension; Diabetes | Hypertension:  PM_2.5_:  Total OR (95%CI): 1.05 (1.05, 1.06);  High FV: 1.08 (1.07, 1.09);  Low FV: 1.03 (1.02, 1.04);  Pint < 0.001;  PM_10_:  Total OR (95%CI): 1.05 (1.05, 1.06);  High FV: 1.07 (1.06, 1.08);  Low FV: 1.04 (1.03, 1.05);  Pint < 0.001;  High meat: 1.06 (1.05, 1.08);  Low meat: 1.05 (1.04, 1.06);  Pint = 0.074;  Diabetes:  PM_2.5_:  Total OR (95%CI): 1.07 (1.06, 1.08);  High FV: 1.09 (1.08, 1.11);  Low FV: 1.05 (1.04, 1.07);  Pint < 0.001;  High meat: 1.07 (1.05, 1.09);  Low meat: 1.07 (1.06, 1.08);  Pint = 0.487;  PM_10_:  Total OR (95%CI): 1.05 (1.04, 1.05);  High FV: 1.06 (1.05, 1.07);  Low FV: 1.04 (1.03, 1.05);  Pint = 0.002;  High meat: 1.04 (1.03, 1.06);  Low meat: 1.05 (1.04, 1.06);  Pint = 0.482; | Age, sex, highest educational attainment, marital status, body mass index, income, Fruits&Vegetables, Meat, Alcohol, Sleep time, Sleep quality, Afternoon naps, Smoke, Secondhand smoke and Physicalactivity |
| Li et al. (2019) | China | — | 18-79 y | Cross-sectional | 39,207 | Fat | PM_1_ | Hypertension | PM_1:_  Total OR (95%CI): 1.04 (1.03,1.05);  High fat: 1.08 (1.06, 1.10);  Low fat: 1.03 (1.02, 1.05);  Pint < 0.001; | Age, sex, marital status, education level, income, smoking, alcohol drinking, physical activity, vegetables and fruits intake, high fat diet, family history of hypertension, body mass index, type 2 diabetes |
| Lin et al. (2017) | China | — | ≥ 50 y | Cross-sectional | 12,665 | Fruits; Vegetables; | PM_2.5_ | Hypertension | PM_2.5:_  Total OR (95%CI): 1.14 (1.07, 1.22);  High fruits: 1.12(1.04, 1.20);  Low fruits: 1.18 (1.09, 1.27);  Pint < 0.05;  High vegetables: 1.13 (1.04, 1.22);  Low vegetables: 1.12 (1.04, 1.21);  Pint > 0.05; | Age, sex, body mass index, consumption of fruit and vegetables, smoking amount, alcohol consumption, physical activity, marital status, urbanity, household income, educational level, domestic fuel type, and ventilation |
| Zhang et al. (2016) | USA | 960,041 person-years | 30-50 y | Cohort | 74,880 | DASH score | PM_2.5_; PM_10_; | Hypertension | PM_2.5_:  Total HR (95%CI): 1.04 (1.00, 1.07);  High DASH score: 1.05 (0.97, 1.14);  Low DASH score: 1.07 (0.99, 1.15);  Pint = 0.570;  PM_10_:  Total HR (95%CI): 1.02 (1.00, 1.04);  High DASH score: 1.03 (0.99, 1.07);  Low DASH score: 1.04 (0.99, 1.08);  Pint = 0.870; | Age, race, calendar year and region, body mass index, DASH diet score, alcohol consumption, smoking status, physical activity, family history of hypertension, menopausal status, nonnarcotic analgesic intake, statin use, diabetes, individual-level socioeconomic status (educational attainment, marital status, partner’s educational attainment, and parental employment), and census-tract median income and home value |
| Zhang et al. (2024) | China | — | ≥ 18 y | Cross-sectional | 3,189 | Traditional southern pattern (TSP) | PM_2.5_ | Hypertension | PM_2.5_:  Total OR (95%CI): 1.42 (1.08, 1.87);  High TSP: 1.18 (0.93, 1.49);  Low TSP: 1.69 (1.29, 2.21);  Pint = 0.044; | Age, BMI, annual household income, the main source of cooking fuel, physical activity level, family history of hypertension, education level, marital status, occupation, smoking habits, alcohol consumption, diabetes mellitus, dyslipidemia and cardiovascular disease |
| Lei et al. (2025) | China | 6.58 y | ≥ 18 y | Cohort | 9,280 | Composite Dietary Antioxidant Index (CDAI) | PM_2.5_ | hypertension | PM_2.5_:  Total HR (95%CI): 1.532(1.410,1.665);  High CDAI: 1.481(1.286,1.706);  Low CDAI: 1.594(1.384,1.835);  Pint = 0.635; | Age, sex, urban-rural residence, ethnicity, educational level, occupation, BMI, smoking, alcohol consumption, leisure physical activity, salt intake, oil intake, diabetes, and dyslipidemia. |
| Zu et al. (2024) | China | — | 18-40 y | Cross-sectional | 9,294 | Empirical dietary inflammatory pattern (EDIP) score | PM_1_; PM_2.5_; PM_10_; | Cardiovascular risk (CVR) | PM_1_:  Total β (95%CI): 0.41 (0.12, 0.96);  High EDIP: 0.78 (0.14, 1.42);  Low EDIP: 0.52 (-0.06, 1.11);  Pint = 0.012;  PM_2.5_:  Totalβ(95%CI): 0.24 (0.15, 0.38);  High EDIP: 0.47 (0.04, 0.86);  Low EDIP: 0.31 (-0.04, 0.79);  Pint = 0.004;  PM_10_:  Total β (95%CI): 0.23 (0.10, 0.36);  High EDIP: 0.48 (0.06, 0.85);  Low EDIP: 0.25 (-0.03, 0.54);  Pint = 0.020; | Age, pre-pregnancy BMI, gestational weight gain,  educational years, family income, parity, residence, family history of diabetes,  family history of hypertension, physical activity, total energy, temperature and  humidity. |
| Zhu et al. (2025) | UK | 13.4 y | 40-69 y | Cohort | 190,092 | Healthy Plant-Based Diet Index (hPDI) | PM_2.5_ | HF | PM_2.5_:  Total HR (95%CI): 1.08 (1.05, 1.12);  High hPDI: 1.19 (1.04, 1.37);  Low hPDI: 1.31 (1.17, 1.47);  Pint = 0.178; | Age and sex and ethnicity, physical activity, education, smoking status, drinking status and BMI |
| Cheng et al. (2025) | UK | 14.3 y | 40-69 y | Cohort | 491,305 | Healthy diet patterns (HDP) | PM_10_; PM_2.5_; NO_2_;  NO_X_; | Ventricular arrhythmias | PM_2.5_:  Total HR (95%CI): 1.08 (1.04, 1.12);  High HDP: 1.06 (1.01, 1.12);  Low HDP: 1.10 (1.01, 1.20);  Pint = 0.320;  PM_10_:  Total HR (95%CI): 1.07 (1.03, 1.11);  High HDP: 1.04 (0.99, 1.10);  Low HDP: 1.11 (1.02, 1.22);  Pint = 0.110;  NO_2_:  Total HR (95%CI): 1.10 (1.06, 1.14);  High HDP: 1.06 (1.01, 1.12);  Low HDP: 1.10 (1.01, 1.20);  Pint = 0.330;  NO_X_:  Total HR (95%CI): 1.09 (1.06, 1.13);  High HDP: 1.04 (0.99, 1.09);  Low HDP: 1.09 (1.01, 1.18);  Pint = 0.130; | Age, sex, ethnicity, education, drinking status, smoking status, healthy diet score, Townsend deprivation index, body mass index, body mass index, physical activity, systolic blood pressure, diastolic blood pressure, hypertension, diabetes mellitus, dyslipidemia, and heart failure |
| Ji et al. (2025) | China | 2 y | ≥ 18 y | Cohort | 5,838,833 | Balanced eating habit (DBI) | PM_1_; PM_10_; O_3_; CO | CVDS | PM_1_:  Total HR (95%CI): 1.37 (1.37, 1.38);  High DBI: 1.36 (1.35, 1.37);  Low DBI: 1.54 (1.51, 1.57);  Pint < 0.001;  PM_10_:  Total HR (95%CI): 1.02 (1.02, 1.02);  High DBI: 1.02 (1.02, 1.02);  Low DBI: 1.02 (1.02, 1.03);  Pint = 0.141;  O_3_:  Total HR (95%CI): 1.41 (1.41, 1.42);  High DBI: 1.39 (1.39, 1.40);  Low DBI: 1.65 (1.62, 1.68);  Pint < 0.001;  CO:  Total HR (95%CI): 1.01 (1.01, 1.01);  High DBI: 1.01 (1.04, 1.01);  Low DBI: 1.02 (1.02, 1.02);  Pint < 0.001; | Age, sex, education, occupation, body mass index, disability condition, CVD family history, eating habits, exercise frequency, smoking status, drinking status, hypertension, and diabetes mellitus |
| Xu (b) et al. (2021) | USA | 9,178,732 person-months | 40-75 y | Cohort | 49,603 | Alternative Healthy Eating Index (AHEI) | PM_2.5_; PM_10_; PM_2.5-10_ | Stroke | PM_2.5_:  Total HR (95%CI): 1.05 (0.88, 1.25);  High AHEI: 1.00 (0.76, 1.31);  Low AHEI: 1.11 (0.84, 1.46);  Pint = 0.850;  PM_2.5-10_:  Total HR (95%CI): 1.04 (0.90, 1.20);  High AHEI: 1.11 (0.90, 1.36);  Low AHEI: 0.98 (0.79, 1.22);  Pint = 0.940;  PM_10_:  Total HR (95%CI): 1.04 (0.93, 1.15);  High AHEI: 1.06 (0.91, 1.23);  Low AHEI: 1.02 (0.87, 1.20);  Pint = 0.940; | Age, race, calendar year, season, Census region, smoking status and pack-years, alcohol consumption, BMI, physical activity, diet quality not including alcohol, family history of CVD, comorbidities, current medication use, marital status, living arrangement, employment status and area-level socioeconomic status, and Census tract population density |
| Chen et al. (2023) | UK | 12.08 years | 39-72 y | Cohort | 179,005 | Omega-3 fatty acids; Omega-6 fatty acids; | PM_2.5_; NO_X_; PM_10_; NO_2_ | Lung cancer | PM_2.5_:  Total HR (95%CI): 2.14 (1.14, 4.01);  High omega-3 fatty acids: 2.65 (1.10, 6.38);  Low omega-3 fatty acids: 1.81 (0.75, 4.36);  High omega-6 fatty acids: 2.84 (1.15, 7.00);  Low omega-6 fatty acids: 1.63 (0.69, 3.86);  NO_X_:  Total OR (95%CI): 1.04 (1.01, 1.08);  High omega-3 fatty acids: 1.04 (0.98, 1.10);  Low omega-3 fatty acids: 1.05 (1.00, 1.10);  High omega-6 fatty acids: 1.04 (0.99, 1.10);  Low omega-6 fatty acids: 1.05 (1.00, 1.10);  PM_10_:  Total OR (95%CI): 0.94 (0.68, 1.31);  High omega-3 fatty acids: 1.21 (0.73, 2.01);  Low omega-3 fatty acids: 0.76 (0.49, 1.16);  High omega-6 fatty acids: 1.24 (0.75, 2.05);  Low omega-6 fatty acids: 0.74 (0.48, 1.14);  NO_2_:  Total OR (95%CI): 1.07 (0.98, 1.16);  High omega-3 fatty acids: 1.08 (0.95, 1.22);  Low omega-3 fatty acids: 1.06 (0.94, 1.19);  High omega-6 fatty acids: 1.06 (0.93, 1.20);  Low omega-6 fatty acids: 1.07 (0.96, 1.20); | Age, sex, education level, Townsend deprivation index, family history of lung cancer, diabetes at baseline, body mass index, pack years of smoking, energy intake, VA retinol equivalents intake, VD intake, fish oil supplementation habit. |
| Chen et al. (2024) | China | 2 years | 2 y | Cohort | 657 | ALA; EPA; DPA; DHA; LA; AA | PM_2.5_ | Allergic rhinitis | PM_2.5_:  Total HR (95%CI): 1.85 (1.16, 2.96);  High ALA: 2.30 (1.10, 4.78);  Low ALA: 1.27 (0.50, 3.21);  *P*int = 0.324;  High EPA: 2.15 (0.91, 5.06);  Low EPA: 2.11 (0.94, 4.76;  *P*int = 0.977;  High DPA: 1.05 (0.42, 2.60)；  Low DPA: 3.02 (1.41, 6.47);  *P*int = 0.081;  High DHA: 2.40 (0.98, 5.87);  Low DHA: 1.69 (0.74, 3.85);  *P*int = 0.576;  High LA: 6.48 (2.27, 18.49);  Low LA: 0.74 (0.32, 1.73);  *P*int = 0.002;  High AA: 2.23 (1.01, 4.91);  Low AA: 1.61 (0.70, 3.68);  *P*int = 0.578; | Maternal age, maternal employment, maternal education, monthly household income, parity, sex, temperature, residential greenness, and NO2. |
| Ma et al. (2025) | UK | 12.76 years | 56.78±8.02 y | Cohort | 82,706 | Omega-3 fatty acids; DHA | PM_2.5_; PM_10_; NO_2_ | COPD | PM_2.5_:  Total HR (95%CI): 1.22 (1.11, 1.33);  High omega-3 fatty acids: 1.07 (0.94, 1.23);  Low omega-3 fatty acids: 1.34 (1.18, 1.53);  *P*int = 0.020;  High DHA: 1.14 (0.99, 1.32);  Low DHA: 1.26 (1.11, 1.42);  *P*int = 0.039;  PM_10_:  Total HR (95%CI): 0.97 (0.79, 1.18);  High omega-3 fatty acids: 0.68 (0.48, 0.97);  Low omega-3 fatty acids: 1.19 (0.93, 1.52);  *P*int = 0.249;  High DHA: 0.84 (0.60, 1.18);  Low DHA: 1.05 (0.82, 1.34);  *P*int = 0.263;  NO_2_:  Total HR (95%CI): 1.62 (1.33, 1.97);  High omega-3 fatty acids: 1.27 (0.92, 1.76);  Low omega-3 fatty acids: 1.90 (1.49, 2.43);  *P*int = 0.001;  High DHA: 1.33 (0.94, 1.87);  Low DHA: 1.81 (1.43, 2.30);  *P*int = 0.007; | Age, sex, ethnicity, BMI, alcohol drinking, qualification, physical activity, and healthy diet score. |
| Wang et al. (2025) | UK | 13.3 years | >18y | Cohort | 162,741 | hPDI | PM_2.5_; NO_2_; NO_X_ | COPD | PM_2.5_:  Total HR (95%CI): 1.10 (1.01, 1.19);  High hPDI: 0.86 (0.72, 1.03);  Low hPDI: 1.41 (1.20, 1.65);  *P*int = 0.001;  NO_2_:  Total HR (95%CI): 1.18 (1.09, 1.29);  High hPDI: 1.02 (0.85, 1.22);  Low hPDI: 1.72 (1.45, 2.03);  *P*int = 0.005;  NO_X_:  Total HR (95%CI): 1.13 (1.04, 1.23);  High hPDI: 1.48 (1.25, 1.74);  Low hPDI: 0.91 (0.76, 1.10);  *P*int = 0.005; | Age, sex, ethnicity, educational level, household income, body mass index, smoking and drinking status. |
| Yang et al. (2025) | UK | — | 57 y | Cross-sectional | 327,147 | Healthy diet score based on the MED model | PM_10_; PM_2.5_; NO_2_; NO_X_; SO_2_; | Lung cancer | PM_10_:  Total HR (95%CI): c;  Healthy diet: 1.23 (1.15, 1.32);  Unhealthy diet: 1.27 (1.17, 1.37);  *P*int = 0.647;  PM_2.5_:  Total HR (95%CI): 1.50 (1.41, 1.59);  Healthy diet: 1.48 (1.37, 1.60);  Unhealthy diet: 1.53 (1.39, 1.67);  *P*int = 0.331;  NO_2_:  Total HR (95%CI): 1.35 (1.29, 1.42);  Healthy diet: 1.35 (1.27, 1.43);  Unhealthy diet: 1.35 (1.26, 1.45);  *P*int = 0.174;  NO_X_:  Total HR (95%CI): 1.29 (1.24, 1.34);  Healthy diet: 1.28 (1.22, 1.35);  Unhealthy diet: 1.29 (1.21, 1.37);  *P*int = 0.264;  SO_2_:  Total HR (95%CI): 1.20 (1.15, 1.26);  Healthy diet: 1.21 (1.14, 1.28);  Unhealthy diet: 1.18 (1.10, 1.28);  *P*int = 0.166; | Age, sex, ethnicity, household income, education level, smoking status, passive smoking, body mass index, healthy diet score, physical activity, alcohol intake, tea intake, family history of lung cancer, time spent outdoor, residential areas, variation in diet, common competing risk diseases, and solid fuel consumption. |
| Andersen et al. (2011) | Denmark | 9.9 years | 56.1(7.6) y | Cohort | 57,053 | Fruits | NO_2_ | COPD | NO_2_:  Total OR (95%CI): 1.08 (1.02, 1.14);  High fruits: 1.04 (0.91, 1.19);  Low fruits: 1.09 (1.02, 1.15);  *P*int = 0.550; | Body mass index, educational level, environmental tobacco smoke, fruit consumption, smoking duration, smoking intensity, smoking status and occupational exposure. |
| Guo et al. (2025) | UK | 13 years | 58 y | Cohort | 120,000 | Dietary Inflammatory Index (DII) | PM_2.5_; PM_10_; NO_2_; NO_X_; SO_2_; CO | COPD; Lung cancer; Diabetes; Stroke; Ischemic heart disease (IHD) | COPD:  PM_2.5_:  Total HR (95%CI): 1.02 (1.00, 1.04);  High DII: 1.04 (1.02, 1.07);  Low DII: 0.98 (0.95, 1.02);  *P*int = 0.008;  PM_10_:  Total HR (95%CI): 1.02 (1.00, 1.03);  High DII: 1.03 (1.01, 1.05);  Low DII: 0.99 (0.97, 1.01);  *P*int = 0.011;  NO_2_:  Total HR (95%CI): 1.01 (1.00, 1.01);  High DII: 1.01 (1.01, 1.02);  Low DII: 1.00 (0.99, 1.01);  *P*int = 0.086;  NO_X_:  Total HR (95%CI): 1.00 (1.00, 1.01);  High DII: 1.01 (1.00, 1.01);  Low DII: 1.00 (1.00, 1.01);  *P*int = 0.066;  SO_2_:  Total HR (95%CI): 1.04 (1.01, 1.06);  High DII: 1.06 (1.02, 1.10);  Low DII: 1.07 (1.02, 1.13);  *P*int = 0.693;  CO:  Total HR (95%CI): 1.00 (1.00, 1.01);  High DII: 1.00 (1.00, 1.01);  Low DII: 1.00 (1.00, 1.00);  *P*int = 0.134;  Lung cancer:  PM_2.5_:  Total HR (95%CI): 1.01 (0.97, 1.05);  High DII: 1.01 (0.95, 1.06);  Low DII: 0.97 (0.90, 1.04);  *P*int = 0.451;  PM_10_:  Total HR (95%CI): 1.02 (0.99, 1.05);  High DII: 1.00 (0.97, 1.04);  Low DII: 0.99 (0.94, 1.04);  *P*int = 0.634;  NO_2_:  Total HR (95%CI): 1.01 (0.99, 1.02);  High DII: 1.01 (0.99, 1.02);  Low DII: 1.00 (0.98, 1.02);  *P*int = 0.921;  NO_X_:  Total HR (95%CI): 1.00 (1.00, 1.01);  High DII: 1.00 (1.00, 1.01);  Low DII: 1.00 (0.99, 1.01);  *P*int = 0.954;  SO_2_:  Total HR (95%CI): 1.01 (0.96, 1.06);  High DII: 0.95 (0.87, 1.05);  Low DII: 0.96 (0.85, 1.08);  *P*int = 0.729;  CO:  Total HR (95%CI): 1.01 (1.00, 1.01);  High DII: 1.00 (1.00, 1.01);  Low DII: 1.00 (1.00, 1.01);  *P*int = 0.973;  Diabetes:  PM_2.5_:  Total HR (95%CI): 1.02 (1.00, 1.04);  High DII: 1.04 (1.01, 1.06);  Low DII: 1.00 (0.97, 1.03);  *P*int = 0.028;  PM_10_:  Total HR (95%CI): 1.02 (1.00, 1.03);  High DII: 1.03 (1.01, 1.05);  Low DII: 1.00 (0.98, 1.02);  *P*int = 0.028;  NO_2_:  Total HR (95%CI): 1.01 (1.00, 1.01);  High DII: 1.02 (1.01, 1.03);  Low DII: 1.00 (0.99, 1.01);  *P*int = 0.001;  NO_X_:  Total HR (95%CI): 1.00 (1.00, 1.01);  High DII: 1.01 (1.01, 1.01);  Low DII: 1.00 (1.00, 1.01);  *P*int = 0.002;  SO_2_:  Total HR (95%CI): 1.03 (1.01, 1.05);  High DII: 1.05 (1.02, 1.09);  Low DII: 1.00 (0.96, 1.05);  *P*int = 0.353;  Stroke:  PM_2.5_:  Total HR (95%CI): 0.98 (0.95, 1.00);  High DII: 1.00 (0.96, 1.03);  Low DII: 0.96 (0.93, 1.00);  *P*int = 0.060;  PM_10_:  Total HR (95%CI): 0.98 (0.97, 1.00);  High DII: 1.00 (0.97, 1.02);  Low DII: 0.97 (0.94, 1.00);  *P*int = 0.055;  NO_2_:  Total HR (95%CI): 0.99 (0.99, 1.00);  High DII: 1.00 (0.99, 1.01);  Low DII: 0.99 (0.98, 1.01);  *P*int = 0.218;  NO_X_:  Total HR (95%CI): 1.00 (0.99, 1.00);  High DII: 1.00 (0.99, 1.00);  Low DII: 1.00 (0.99, 1.00);  *P*int = 0.245;  SO_2_:  Total HR (95%CI): 1.05 (1.02, 1.08);  High DII: 1.07 (1.02, 1.13);  Low DII: 1.05 (1.00, 1.11);  *P*int = 0.420;  CO:  Total HR (95%CI): 1.00 (0.99, 1.00);  High DII: 1.00 (1.00, 1.00);  Low DII: 1.00 (1.00, 1.00);  *P*int = 0.394;  IHD:  PM_2.5_:  Total HR (95%CI): 1.00 (0.98, 1.01);  High DII: 1.01 (0.99, 1.03);  Low DII: 1.00 (0.99, 1.02);  *P*int = 0.947;  PM_10_:  Total HR (95%CI): 1.00 (0.99, 1.01);  High DII: 1.00 (0.99, 1.02);  Low DII: 1.00 (0.99, 1.01);  *P*int = 0.995;  NO_2_:  Total HR (95%CI): 1.00 (1.00, 1.00);  High DII: 1.00 (0.99, 1.01);  Low DII: 1.00 (0.99, 1.01);  *P*int = 0.871;  NO_X_:  Total HR (95%CI): 1.00 (1.00, 1.00);  High DII: 1.00 (1.00, 1.00);  Low DII: 1.00 (1.00, 1.00);  *P*int = 0.797;  SO_2_:  Total HR (95%CI): 1.03 (1.01, 1.05);  High DII: 1.06 (1.03, 1.09);  Low DII: 1.02 (0.99, 1.05);  *P*int = 0.005;  CO:  Total HR (95%CI): 1.00 (1.00, 1.00);  High DII: 1.00 (1.00, 1.00);  Low DII: 1.00 (1.00, 1.00);  *P*int = 0.897; | Age, sex, region, annual household income, current employment status, qualifications, ethnicity, activity intensity, BMI, smoking, dietary inflammatory index, and comorbidities and family history specific to each event |
| Lamichhane et al. (2017) | Korean | — | 60.8 (10.4) y | Case-Control | 908 pairs | Fruits | PM_10_; NO_2_ | Lung cancer | PM_10_:  Total OR (95%CI): 1.09 (0.96, 1.23);  High fruits: 1.01 (0.77, 1.31);  Low fruits: 1.44 (1.06, 1.96);  *P*int = 0.196;  NO_2_:  Total OR (95%CI): 1.10 (1.00, 1.22);  High fruits: 1.20 (0.98, 1.46);  Low fruits: 1.03 (0.80, 1.32);  *P*int = 0.600; | Age, sex, smoking, education, exposure to occupational carcinogens, and frequency of fruit intake |
| Ole et al. (2011) | Denmark | 9.6 years | 50–64 y | Cohort | 52,970 | Fruits | NO_X_ | Lung cancer | NO_X_:  Total RR (95%CI): 1.09 (0.79, 1.51);  High fruits: 0.89 (0.54, 1.45);  Low fruits: 1.26 (0.82, 1.56);  *P*int = 0.670; | Smoking, ETS, length of school attendance, fruit intake, and employment |
| Fan et al. (2024) | UK | 12.19 years | 40-70 y | Cohort | 78,230 | MED; DASH diet; Anti-EDIP; AONDP; Anti-PONDP; AODP | PM_2.5_; PM_10_; NO_2_; NO_X_ | Type 2 diabetes | PM_2.5_:  Total HR (95%CI): 1.07 (1.01, 1.14);  High MED: 1.06 (1.00, 1.12);  Low MED: 1.11 (1.05, 1.18);  Pint = 0.448;  High DASH diet: 1.05 (0.99, 1.12);  Low DASH diet: 1.12 (1.05, 1.18);  Pint = 0.401;  High anti-EDIP: 1.06 (1.00, 1.13);  Low anti-EDIP: 1.11 (1.05, 1.18);  Pint = 0.481;  High AONDP:1.08 (1.02, 1.14);  Low AONDP: 1.10 (1.03, 1.16);  Pint = 0.910;  High anti-PONDP: 1.07 (1.00, 1.13);  Low anti-PONDP: 1.10 (1.04, 1.17);  Pint = 0.698;  High AODP: 1.05 (0.99, 1.12);  Low AODP: 1.12 (1.06, 1.19);  Pint = 0.328;  PM_10_:  Total HR (95%CI): 1.03 (0.99, 1.08);  High MED: 1.02 (0.98, 1.07);  Low MED: 1.07 (1.02, 1.12);  Pint = 0.355;  High DASH diet: 1.02 (0.97, 1.07);  Low DASH diet: 1.07 (1.02, 1.12);  Pint = 0.309;  High anti-EDIP: 1.03 (0.98, 1.08);  Low anti-EDIP: 1.07 (1.02, 1.12);  Pint = 0.381;  High AONDP: 1.04 (0.99, 1.09);  Low AONDP: 1.05 (1.01, 1.10);  Pint = 0.892;  High anti-PONDP: 1.03 (0.98, 1.07);  Low anti-PONDP: 1.06 (1.01, 1.10);  Pint = 0.651;  High AODP: 1.02 (0.97, 1.07);  Low AODP: 1.07 (1.03, 1.12);  Pint = 0.269;  NO_2_:  Total HR (95%CI): 1.10 (1.03, 1.17);  High MED: 1.03 (0.96, 1.10);  Low MED: 1.18 (1.10, 1.27);  Pint = 0.010;  High DASH diet: 1.03 (0.96, 1.10);  Low DASH diet: 1.20 (1.13, 1.28);  Pint = 0.005;  High anti-EDIP: 1.05 (0.98, 1.13);  Low anti-EDIP: 1.20 (1.12, 1.28);  Pint = 0.008;  High AONDP: 1.08 (1.01, 1.16);  Low AONDP: 1.14 (1.07, 1.22);  Pint = 0.578;  High anti-PONDP: 1.05 (0.97, 1.12);  Low anti-PONDP: 1.16 (1.08, 1.24);  Pint = 0.120;  High AODP: 1.01 (0.92, 1.10);  Low AODP: 1.21 (1.12, 1.31);  Pint = 0.007;  NO_X_:  Total HR (95%CI): 1.07 (1.02, 1.12);  High MED: 1.00 (0.95, 1.06);  Low MED: 1.14 (1.08, 1.21);  Pint = 0.002;  High DASH diet: 1.00 (0.95, 1.06);  Low DASH diet: 1.16 (1.10, 1.22);  Pint = 0.001;  High anti-EDIP: 1.02 (0.97, 1.08);  Low anti-EDIP: 1.16 (1.10, 1.21);  Pint = 0.001;  High AONDP: 1.06 (1.00, 1.12);  Low AONDP: 1.10 (1.05, 1.16);  Pint = 0.507;  High anti-PONDP: 1.02 (0.96, 1.08);  Low anti-PONDP: 1.12 (1.06, 1.18);  Pint = 0.048;  High AODP: 0.98 (0.91, 1.06);  Low AODP: 1.16 (1.09, 1.23);  Pint = 0.003; | Age, sex, ethnicity, employment status, education level, average total household income, smoking status, alcohol status, physical activity, and polygenic risk score. |
| Fan et al. (2025) | UK | 12.19 years | 40-70 y | Cohort | 77,278 | Total beverage score (TBS) | PM_2.5_; PM_10_; NO_2_; NO_X_ | Type 2 diabetes | PM_2.5_:  Total HR (95%CI): 1.09 (1.03, 1.16);  High TBS: 1.03 (0.97, 1.10);  Low TBS: 1.13 (1.06, 1.20);  Pint = 0.256;  PM_10_:  Total HR (95%CI): 1.05 (1.00, 1.10);  High TBS: 1.00 (0.95, 1.05);  Low TBS: 1.08 (1.03, 1.13);  Pint = 0142;  NO_2_:  Total HR (95%CI): 1.14 (1.06, 1.21);  High TBS: 0.97 (0.90, 1.06);  Low TBS: 1.25 (1.17, 1.35);  Pint < 0.001;  NO_X_:  Total HR (95%CI): 1.09 (1.04, 1.15);  High TBS: 0.93 (0.87, 1.00);  Low TBS: 1.18 (1.12, 1.24);  Pint < 0.001; | Age, sex, ethnicity, residential area, employment status, education level, house income, dietary energy intake, smoking status, alcohol status, weekly physical activity, systolic blood pressure, diastolic blood pressure, polygenic risk score for T2D, dietary approaches to stop hypertension diet score, and DASH score. |
| Margetaki et al. (2024) | Greece | 6.5 years | 4.2/6.5 y | Cohort | 633 pairs | FV | PM_2.5_; PM_10_ | Obesity; Abdominal obesity | Obesity (4 y):  PM_2.5_:  Total RR (95%CI): 1.11 (0.76, 1.63);  High FV: 0.99 (0.65, 1.52);  Low FV: 1.35 (0.81, 2.24);  Pint: 0.289;  PM_10_:  Total RR (95%CI): 0.83 (0.55, 1.24);  High FV: 0.64 (0.37, 1.13);  Low FV: 1.13 (0.66, 1.92);  Pint: 0.138;  Obesity (6 y):  PM_2.5_:  Total RR (95%CI): 1.10 (0.83, 1.45);  High FV: 0.77 (0.47, 1.27);  Low FV: 1.67 (1.09, 2.54);  Pint: 0.023;  PM_10_:  Total RR (95%CI): 1.10 (0.83, 1.45);  High FV: 0.77 (0.47, 1.27);  Low FV: 1.67 (1.09, 2.54);  Pint: 0.023;  Abdominal obesity (4 y):  PM_2.5_:  Total RR (95%CI): 0.90 (0.69, 1.19);  High FV: 0.85 (0.63, 1.15);  Low FV: 1.04 (0.67, 1.61);  Pint: 0.401;  PM_10_:  Total RR (95%CI): 0.81 (0.59, 1.10);  High FV: 0.78 (0.56, 1.07);  Low FV: 0.88 (0.47, 1.64);  Pint: 0.715;  Abdominal obesity (6 y):  PM_2.5_:  Total RR (95%CI): 1.21 (0.92, 1.60);  High FV: 0.94 (0.67, 1.32);  Low FV: 1.90 (1.08, 3.35);  Pint: 0.039;  PM_10_:  Total RR (95%CI): 1.08 (0.78, 1.48);  High FV: 0.71 (0.41, 1.24);  Low FV: 1.68 (1.02, 2.79);  Pint: 0.028; | Age, sex, maternal education, household disposable income, maternal age, pre-pregnancy body mass index, maternal smoking during pregnancy, degree of urbanicity during pregnancy, season of birth, and child screen time and adherence to the Mediterranean diet. |
| Shin et al. (2023) | Korean | 5 years | 58.6 (9.4) y | Cohort | 14,667 | Retinol; Vitamin A (VA); Mini dietary assessment index score (MDAIS); Milk; Vegetables; Fruits | PM_2.5_; NO_2_ | Diabetes | PM_2.5_:  Total HR (95%CI): 1.49 (1.11, 2.00);  High retinol: 2.35 (1.30, 4.23);  Low retinol: 1.63 (0.98, 2.74);  Pint: 0.38;  High VA: 1.38 (0.86, 2.24);  Low VA: 1.44 (0.83, 2.52);  Pint: 0.80;  High MDAIS: 1.22 (0.87, 1.70);  Low MDAIS: 1.70 (1.05, 2.77);  Pint: 0.64;  High milk: 1.44 (0.93, 2.22);  Low milk: 1.48 (1.02, 2.16);  Pint: 0.66;  High vegetables: 1.47 (1.01, 2.15);  Low vegetables: 1.42 (0.98, 2.07);  Pint: 0.61;  High fruits: 1.92 (1.27, 2.90);  Low fruits: 1.16 (0.82, 1.63);  Pint: 0.14;  NO_2_:  Total HR (95%CI): 1.29 (1.12, 1.49);  High retinol: 1.21 (0.89, 1.64);  Low retinol: 1.65 (1.32, 2.07);  Pint: 0.01;  High VA: 1.21 (0.94, 1.56);  Low VA: 1.76 (1.33, 2.33);  Pint: 0.04;  High MDAIS: 1.18 (1.00, 1.40);  Low MDAIS: 1.46 (1.14, 1.88);  Pint: 0.19;  High milk: 1.20 (0.94, 1.52);  Low milk: 1.39 (1.15, 1.67);  Pint: 0.22;  High vegetables: 1.20 (1.01, 1.42);  Low vegetables: 1.34 (1.11, 1.63);  Pint: 0.28;  High fruits: 1.36 (1.12, 1.64);  Low fruits: 1.20 (1.00, 1.43);  Pint: 0.34; | Sex, monthly household income, educational levels, cigarette smoking, alcohol consumption, regular physical activity, and body mass index, with a random effect of survey sites. |
| Li (b) et al. (2022) | UK | 11.7 years | 55.78 (7.98) y | Cohort | 156,490 | VA; VC; VE | PM_2.5_; PM_10_; NO_2_; NOx | Diabetes | PM_2.5_:  Total HR (95%CI): 1.50 (1.29, 1.73);  High VA: 1.18 (0.89, 1.57);  Low VA: 1.53 (1.29, 1.81);  PM_10_:  Total HR (95%CI): 1.15 (1.07, 1.24);  High VA: 1.06 (0.91, 1.22);  Low VA: 1.13 (1.04, 1.24);  NO_2_:  Total HR (95%CI): 1.04 (1.02, 1.05);  High VA: 1.01 (0.98, 1.04);  Low VA: 1.04 (1.02, 1.06);  NO_X_:  Total HR (95%CI): 1.02 (1.02, 1.03);  High VA: 1.01 (1.00, 1.03);  Low VA: 1.02 (1.01, 1.04); | Age, sex, ethnicity, education attainment, average household income, waist to hip ratio, physical activity, smoking status, alcohol intake, vitamin supplement, protein intake, polyunsaturated fat intake, total sugar intake and fiber intake in diet. |
| Zheng et al. (2024) | UK | 10.1 years | 55.7 (8.0) y | Cohort | 162,579 | Dietary diversity score (DDS) | PM_2.5_; PM_10_; SO_2_; NO_2_; NOx | Type 2 diabetes | PM_2.5_:  Total HR (95%CI): 1.08 (1.03, 1.14);  High DDS: 1.03 (0.92, 1.15);  Low DDS: 1.23 (1.10, 1.38);  Pint: 0.026;  PM_10_:  Total HR (95%CI): 1.06 (1.01, 1.12);  High DDS: 1.01 (0.91, 1.13);  Low DDS: 1.20 (1.07, 1.35);  Pint: 0.016;  SO_2_:  Total HR (95%CI): 1.10 (1.05,1.15);  High DDS: 1.08 (0.99, 1.18);  Low DDS: 1.16 (1.05, 1.29);  Pint: 0.995;  NO_2_:  Total HR (95%CI): 1.07 (1.02, 1.12);  High DDS: 0.96 (0.86, 1.07);  Low DDS: 1.24 (1.12, 1.38);  Pint: 0.003;  NO_X_:  Total HR (95%CI): 1.06 (1.01, 1.11);  High DDS: 0.96 (0.87, 1.06);  Low DDS: 1.22 (1.11, 1.34);  Pint: 0.005; | Age, sex, race, household income, residence, family history of diabetes, smoking status, alcohol consumption, physical activity, BMI, multivitamin supplement, CVD, cancer, hypertension, hyperlipemia, DDS, and total energy intake. |
| Guo et al. (2020) | China | — | 6–17 y | Cross-sectional | 41,439 | Vegetables; Fruits; | PM_2.5_ | Obesity | PM_2.5_:  Total OR (95%CI): 1.10 (1.03, 1.16);  High vegetables: 1.05 (1.03, 1.08);  Low vegetables: 1.05 (1.03, 1.08);  Pint: 0.679;  High fruits: 1.05 (1.03, 1.08);  Low fruits: 1.04 (1.02, 1.07);  Pint: 0.135; | Age, sex, urbanity, boarding school or not, economic level, mother’s occupation level, mother’s educational level, vegetable intake, fruit intake, beverages intake, activity time, household cooking fuel type, household heating fuel type, school heating fuel type, home ventilation time, and secondhand smoke |
| Liu et al. (2020) | China | — | 18–79 y | Cross-sectional | 38,824 | FV; fat | PM_1_; PM_2.5_; PM_10_; NO_2_; | Obesity (BMI); Obesity (WC);  Obesity (WHR); Obesity (WHtR); Obesity (BFP); Obesity (VFI); | Obesity (BMI):  PM_1_:  Total OR (95%CI): 1.18 (1.14, 1.22);  High FV: 1.19 (1.12, 1.27);  Low FV: 1.18 (1.13, 1.23);  High fat: 1.18 (1.10, 1.27);  Low fat: 1.18 (1.14, 1.23);  PM_2.5_:  Total OR (95%CI): 1.42 (1.35, 1.49);  High FV: 1.48 (1.37, 1.60);  Low FV: 1.38 (1.29, 1.47);  High fat: 1.37 (1.24, 1.52);  Low fat: 1.43 (1.36, 1.52);  PM_10_:  Total OR (95%CI): 1.51 (1.43, 1.59);  High FV: 1.57 (1.45, 1.70);  Low FV: 1.48 (1.38, 1.58);  High fat: 1.43 (1.28, 1.60);  Low fat: 1.54 (1.45, 1.64);  NO_2_:  Total OR (95%CI): 1.42 (1.35, 1.49);  High FV: 1.53 (1.41, 1.65);  Low FV: 1.37 (1.28, 1.46);  High fat: 1.36 (1.23, 1.51);  Low fat: 1.44 (1.36, 1.53);  Obesity (WC):  PM_1_:  Total OR (95%CI): 1.26 (1.22, 1.30);  High FV: 1.30 (1.24, 1.37);  Low FV: 1.22 (1.18, 1.26);  High fat: 1.22 (1.15, 1.30);  Low fat: 1.27 (1.23, 1.31);  PM_2.5_:  Total OR (95%CI): 1.56 (1.50, 1.62);  High FV: 1.66 (1.56, 1.77);  Low FV: 1.47 (1.40, 1.54);  High fat: 1.47 (1.35, 1.60);  Low fat: 1.59 (1.52, 1.66);  PM_10_:  Total OR (95%CI): 1.76 (1.69, 1.84);  High FV: 1.82 (1.70, 1.94);  Low FV: 1.70(1.61, 1.79);  High fat: 1.62 (1.48, 1.77);  Low fat: 1.81 (1.73, 1.90);  NO_2_:  Total OR (95%CI): 1.72 (1.65, 1.79);  High FV: 1.80 (1.68, 1.92);  Low FV: 1.65 (1.57, 1.74);  High fat: 1.54 (1.42, 1.68);  Low fat: 1.78 (1.70, 1.86);  Obesity (WHR):  PM_1_:  Total OR (95%CI): 1.05 (1.02, 1.08);  High FV: 1.04 (0.99, 1.09);  Low FV: 1.04(1.00, 1.08);  High fat: 1.08 (1.02, 1.15);  Low fat: 1.04 (1.01, 1.08);  PM_2.5_:  Total OR (95%CI): 1.18 (1.14, 1.23);  High FV: 1.18 (1.11, 1.26);  Low FV: 1.16 (1.10, 1.22);  High fat: 1.21 (1.11, 1.32);  Low fat: 1.18 (1.13, 1.23);  PM_10_:  Total OR (95%CI): 1.35 (1.29, 1.41);  High FV: 1.30 (1.22, 1.39);  Low FV: 1.35 (1.28, 1.42);  High fat: 1.32 (1.21, 1.45);  Low fat: 1.36 (1.30, 1.43);  NO_2_:  Total OR (95%CI): 1.36 (1.30, 1.41);  High FV: 1.32 (1.24, 1.41);  Low FV: 1.35 (1.28, 1.42);  High fat: 1.29 (1.18, 1.40);  Low fat: 1.38 (1.32, 1.44);  Obesity (WHtR):  PM_1_:  Total OR (95%CI): 1.27 (1.23, 1.31);  High FV: 1.30 (1.24, 1.37);  Low FV: 1.22 (1.17, 1.26);  High fat: 1.27 (1.19, 1.36);  Low fat: 1.27 (1.22, 1.31);  PM_2.5_:  Total OR (95%CI): 1.58 (1.52, 1.65);  High FV: 1.65 (1.54, 1.77);  Low FV: 1.49 (1.41, 1.57);  High fat: 1.56 (1.42, 1.71);  Low fat: 1.59 (1.52, 1.67);  PM_10_:  Total OR (95%CI): 1.78 (1.70, 1.87);  High FV: 1.80 (1.68, 1.93);  Low FV: 1.71 (1.61, 1.81);  High fat: 1.73 (1.58, 1.91);  Low fat: 1.80 (1.71, 1.90);  NO_2_:  Total OR (95%CI): 1.72 (1.65, 1.79);  High FV: 1.78 (1.66, 1.91);  Low FV: 1.63 (1.55, 1.72);  High fat: 1.65 (1.51, 1.81);  Low fat: 1.74 (1.66, 1.83);  Obesity (BFP):  PM_1_:  Total OR (95%CI): 1.06 (1.03, 1.09);  High FV: 1.03 (0.98–1.09);  Low FV: 1.06 (1.02, 1.10);  High fat: 1.10 (1.03–1.18);  Low fat: 1.05 (1.01, 1.09);  PM_2.5_:  Total OR (95%CI): 1.30 (1.24, 1.36);  High FV: 1.26 (1.17, 1.35);  Low FV: 1.29 (1.22, 1.36);  High fat: 1.33 (1.21, 1.46);  Low fat: 1.29 (1.22, 1.36);  PM_10_:  Total OR (95%CI): 1.43 (1.36, 1.50);  High FV: 1.37 (1.28, 1.48);  Low FV: 1.43 (1.34, 1.52);  High fat: 1.44 (1.30–1.58);  Low fat: 1.43 (1.35, 1.51);  NO_2_:  Total OR (95%CI): 1.34 (1.28, 1.40);  High FV: 1.36 (1.27, 1.46);  Low FV: 1.31 (1.23, 1.38);  High fat: 1.38 (1.26, 1.51);  Low fat: 1.33 (1.26, 1.40);  Obesity (VFI):  PM_1_:  Total OR (95%CI): 1.15 (1.12, 1.19);  High FV: 1.16 (1.10, 1.21);  Low FV: 1.14 (1.10, 1.18);  High fat: 1.19 (1.12, 1.27);  Low fat: 1.14 (1.11, 1.18);  PM_2.5_:  Total OR (95%CI): 1.39 (1.33, 1.45);  High FV: 1.41 (1.32, 1.51);  Low FV: 1.36 (1.29, 1.43);  High fat: 1.42 (1.30, 1.55);  Low fat: 1.38 (1.32, 1.45);  PM_10_:  Total OR (95%CI): 1.48 (1.41, 1.54);  High FV: 1.49 (1.39, 1.59);  Low FV: 1.46 (1.38, 1.55);  High fat: 1.53 (1.39, 1.68);  Low fat: 1.47 (1.40, 1.54);  NO_2_:  Total OR (95%CI): 1.38 (1.33, 1.44);  High FV: 1.45 (1.36, 1.55);  Low FV: 1.34 (1.27, 1.41);  High fat: 1.44 (1.32, 1.58);  Low fat: 1.37 (1.31, 1.43); | Age, sex, education level, per capita monthly income, smoking, drinking, fat diet, fruit and vegetable intake, and physical activity. |
| Yang (a) et al. (2018) | China | — | 50 y | Cross-sectional | 11,504 | Vegetables; Fruits; | PM_2.5_ | Diabetes | PM_2.5_:  Total OR (95%CI): 1.27 (1.12, 1.43);  High vegetables: 1.24 (1.07, 1.44);  Low vegetables: 1.18 (1.04, 1.34);  *P*int = 0.230;  High fruits: 1.19 (1.08, 1.30);  Low fruits: 1.47 (1.21, 1.78);  *P*int = 0.001; | Age, sex, BMI, smoking, education, household income, physical activity, residence, and occupational pollution exposure |
| Mao et al. (2020) | China | — | 18–79 y | Cross-sectional | 39,057 | FV; fat | PM_1_ | DLP; HCH; HTG; HALP; HBLP | DLP:  PM_1_:  Total OR (95%CI): 0.99 (0.97, 0.99);  High FV: 0.98 (0.96, 0.99);  Low FV: 1.00 (0.98, 1.01);  Pint = 0.092;  High fat: 1.06 (0.98, 1.03);  Low fat: 0.99 (0.97, 0.99);  Pint = 0.112;  HCH:  PM_1_:  Total OR (95%CI): 1.06 (1.04, 1.08);  High fat: 1.09 (1.04, 1.14);  Low fat: 1.06 (1.04, 1.08);  Pint = 0.278;  HTG:  PM_1_:  Total OR (95%CI): 0.92 (0.91, 0.93);  High FV: 0.90 (0.88, 0.92);  Low FV: 0.95 (0.93, 0.96);  Pint < 0.001;  High fat: 0.95 (0.92, 0.97);  Low fat: 0.92 (0.91, 0.94);  Pint = 0.096;  HALP:  PM_1_:  Total OR (95%CI): 1.05 (1.03, 1.07);  High FV: 1.04 (1.02, 1.06);  Low FV: 1.04 (1.02, 1.06);  Pint = 0.859;  High fat: 1.04 (1.01, 1.07);  Low fat: 1.04 (1.02, 1.05);  Pint = 0.835;  HBLP:  PM_1_:  Total OR (95%CI): 1.03 (1.02, 1.05);  High FV: 1.06 (1.02, 1.09);  Low FV: 1.05 (1.02, 1.08);  Pint = 0.692;  High fat: 1.05 (1.00, 1.10);  Low fat: 1.05 (1.03, 1.07);  Pint = 0.906; | Age, sex, BMI, education, marital status, family income, smoking, alcohol drinking, high fat diet, adequate vegetable and fruit intake, physical activities and family history of dyslipidemia |
| Wang et al. (2022) | China | — | ≥ 18 y | Cross-sectional | 10,253 | Fruits | NO_2_; SO_2_; PM_10_; PM_2.5_; O_3_; | Metabolic  Syndrome (MS) | NO_2_:  Total OR (95%CI): 0.87 (0.80, 0.94);  High fruits: 0.87 (0.79, 0.94);  Low fruits: 0.86 (0.73, 0.99);  Pint = 0.951;  SO_2_:  Total OR (95%CI): 0.88 (0.78, 1.00);  High fruits: 0.87 (0.76, 0.98);  Low fruits: 0.92 (0.72, 1.13);  Pint = 0.677;  PM_10_:  Total OR (95%CI): 1.22 (1.07, 1.39);  High fruits: 1.15 (0.99, 1.30);  Low fruits: 1.57 (1.18, 1.97);  Pint = 0.048;  PM_2.5_:  Total OR (95%CI): 1.19 (1.03, 1.39);  High fruits: 1.12 (0.95, 1.29);  Low fruits: 1.59 (1.16, 2.02);  Pint = 0.048;  O_3_:  Total OR (95%CI): 1.07 (1.03, 1.12);  High fruits: 1.08 (1.02, 1.13);  Low fruits: 1.06 (0.97, 1.15);  Pint = 0.754; | Age, sex, employment, marital status, education level, annual family income, drinking, smoking status, vegetable intake, fruit intake, family fuel type and region |
| Yang (b) et al. (2018) | China | — | 18–74 y | Cross-sectional | 15,477 | SSB; Controlled diet of low calories and low fat (LCD-LFD) | PM_1_; NO_2_; SO_2_; PM_10_; PM_2.5_; O_3_; | MS | PM_1_:  Total OR (95%CI): 1.12 (1.00, 1.24);  High SSB: 1.21 (1.14, 1.28);  Low SSB: 1.12 (1.08, 1.16);  Pint > 0.05;  High LCD-LFD: 1.09 (1.02, 1.16);  Low LCD-LFD: 1.14 (1.09, 1.18);  Pint > 0.05;  NO_2_:  Total OR (95%CI): 1.33 (1.12, 1.57);  High SSB: 1.56 (1.40, 1.74);  Low SSB: 1.36 (1.27, 1.45);  Pint > 0.05;  High LCD-LFD: 1.21 (1.06, 1.37);  Low LCD-LFD: 1.42 (1.32, 1.53);  Pint < 0.05;  SO_2_:  Total OR (95%CI): 1.10 (1.02, 1.18);  High SSB: 1.19 (1.20, 1.25);  Low SSB: 1.11 (1.08, 1.14);  Pint > 0.05;  High LCD-LFD: 1.10 (1.07, 1.13);  Low LCD-LFD: 1.14 (1.08, 1.20);  Pint > 0.05;  PM_10_:  Total OR (95%CI): 1.13 (1.08, 1.19);  High SSB: 1.18 (1.14, 1.23);  Low SSB: 1.14 (1.11, 1.17);  Pint > 0.05;  High LCD-LFD: 1.11 (1.05, 1.16);  Low LCD-LFD: 1.15 (1.12, 1.18);  Pint > 0.05;  PM_2.5_:  Total OR (95%CI): 1.09 (1.00, 1.18);  High SSB: 1.14 (1.09, 1.19);  Low SSB: 1.07 (1.05, 1.10);  Pint > 0.05;  High LCD-LFD: 1.06 (1.01, 1.11);  Low LCD-LFD: 1.08 (1.05, 1.11);  Pint > 0.05;  O_3_:  Total OR (95%CI): 1.10 (1.01, 1.18);  High SSB: 1.20 (1.12, 1.27);  Low SSB: 1.10 (1.07, 1.13);  Pint > 0.05;  High LCD-LFD: 1.11 (1.05, 1.17);  Low LCD-LFD: 1.10 (1.07, 1.14);  Pint > 0.05; | Age, sex, income, education, career, smoking status, alcohol consumption, regular exercise, controlled diet of low calories and low fat, sugar-sweetened soft drink intake, and district |
| Zheng et el. (2022) | China | — | 50.12 (14.73) y | Cross-sectional | 6,628 | Grain; FV; Red Meat | PM_2.5_ | MS; HTG | MS:  PM_2.5_:  Total OR (95%CI): 1.17 (1.01, 1.35);  High grain: 1.09 (0.90, 1.34);  Low grain: 1.32 (1.08, 1.62);  Pint = 0.713;  High FV: 1.14 (0.92, 1.41);  Low FV: 1.25 (1.03, 1.51);  Pint = 0.979;  High red meat: 1.19 (0.96, 1.49);  Low red meat: 1.16 (0.97, 1.39);  Pint = 0.312;  HTG:  PM_2.5_:  Total OR (95%CI): 1.36 (1.18, 1.58);  High grain: 1.33 (1.11, 1.61);  Low grain: 1.38 (1.14, 1.68);  Pint = 0.897;  High FV: 1.36 (1.10, 1.66);  Low FV: 1.41 (1.18, 1.69);  Pint = 0.869;  High red meat: 1.38 (1.13, 1.70);  Low red meat: 1.29 (1.09, 1.53);  Pint = 0.332; | Age, sex, education, marital status, body mass index, household income, exercise, cigarette smoking status, biomass fuel, alcohol consumption, red meat consumption |
| Li (a) et al. (2023) | China | 5 weeks | 21.09 (1.08) y | Cohort | 88 | Vitamin D (VD) | PM_2.5_, PM_1_, PM_0.5_, | HDL-C; LDL-C; TC; TGs; | HDL-C:  PM_2.5_:  High VD: -1.98% (-7.47%, 3.83%);  Low VD: -4.33% (-7.83%, -0.70%);  Pint = 0.335;  PM_1_:  High VD: -1.96% (-7.33%, 3.72%);  Low VD: -4.26% (-7.69%, -0.71%);  Pint = 0.332;  PM_0.5_:  High VD: -1.76% (-6.79%, 3.54%);  Low VD: -3.86% (-7.01%, -0.59%);  Pint = 0.346;  LDL-C:  PM_2.5_:  High VD: -0.31% (-7.53%, 7.48%);  Low VD: -2.67% (-7.29%, 2.19%);  Pint = 0.466;  PM_1_:  High VD: -0.21% (-7.29%, 7.41%);  Low VD: -2.45% (-6.99%, 2.30%);  Pint = 0.477;  PM_0.5_:  High VD: -0.23% (-6.85%, 6.86%);  Low VD: -2.25% (-6.42%, 2.11%);  Pint = 0.495;  TC:  PM_2.5_:  High VD: -0.10% (-4.53%, 4.53%);  Low VD: 0.21% (-2.69%, 3.20%);  Pint = 0.873;  PM_1_:  High VD: -0.02% (-4.36%, 4.52%);  Low VD: 0.27% (-2.57%, 3.19%);  Pint = 0.881;  PM_0.5_:  High VD: -0.05% (-4.11%, 4.17%);  Low VD: 0.32% (-2.28%, 2.99%);  Pint = 0.837;  TGs:  PM_2.5_:  High VD: -0.20% (-23.23%, 29.74%);  Low VD: 17.91% (-0.55%, 39.80%);  Pint = 0.144;  PM_1_:  High VD: 0.20% (-22.49%, 29.53%);  Low VD: 16.89% (-1.06%, 38.08%);  Pint = 0.166;  PM_0.5_:  High VD: -0.01% (-21.30%, 27.05%);  Low VD: 15.67% (-0.69%, 34.73%);  Pint = 0.160; | Age, gender, body mass index, exercise, dietary fat intake, time trend, and temperature |
| Cao et al. (2024) | China | — | 6 to 17 y | Cross-sectional | 3,711 | Sodium | PM_2.5_ | HDL-C; LDL-C; TC; TGs; | HDL-C:  PM_2.5_:  Total change% (95%CI): -1.49% (-2.35%, -0.62%);  High sodium: -2.35% (-4.07%, -0.60%);  Low sodium: -0.79% (-2.55%, -1.00%);  Pint = 0.0023;  LDL-C:  PM_2.5_:  Total change% (95%CI): 2.26% (1.15%, 3.38%);  High sodium: 2.6% (0.26%, 5.00%);  Low sodium: -0.58% (-2.66%, 1.54%);  Pint = 0.0121;  TC:  PM_2.5_:  Total change% (95%CI): 1.56% (0.90%, 2.23%);  High sodium: 1.42% (0.04%, 2.83%);  Low sodium: 0.27% (-1.02%, 1.57%);  Pint = 0.0025;  TG:  PM_2.5_:  Total change% (95%CI): 0.08% (-1.43%, 1.62%);  High sodium: 2.15% (-0.92%, 5.31%);  Low sodium: -3.08% (-5.90%, -0.17%);  Pint = 0.0549; | Region, father’s ethnicity, father’s occupation, father’s education, child’s age, child’s sex, child’s BMI, childhood daily average outdoor physical activity duration and passive smoking |
| Zu et al. (2023) | China | — | 35.04 ±3.6 y | Cross-sectional | 8495 pregnant women | Empirical dietary inflammatory pattern (EDIP) score | PM_2.5;_  PM_10;_  SO_2;_ | Gestational diabetes mellitus | PM_2.5_:  Total OR (95%CI): 1.18 (1.11, 1.25);  High EDIP: 1.12 (1.01, 1.25);  Low EDIP: 1.10 (0.98, 1.23);  Pint = 0.009;  PM_10_:  Total OR (95%CI): 1.15 (1.10, 1.19);  High EDIP: 1.10 (1.03, 1.38);  Low EDIP: 1.06 (0.95, 1.19);  Pint = 0.031;  SO_2_:  Total OR (95%CI): 1.50 (1.34, 1.70);  High EDIP: 1.43 (1.08, 2.05);  Low EDIP: 1.27 (0.95, 2.10);  Pint = 0.012; | Age, maternal education, income, pre-pregnancy BMI, systolic blood pressure, husband’s education, parity, season of blood collection, family history of diabetes, physical activity, passive smoking, fruit intake, and temperature |
| Zhang et al. (2021) | China | 3 years | 31.67y | Cohort | 2,244 | HDP; Sweet foods pattern (SFP); Animal foods pattern (AFP); Traditional pattern (TP); | PM_2.5;_ PM_10;_ SO_2;_ NO_2;_ CO; O_3_ | Gestational diabetes mellitus | PM_2.5_:  Total OR (95%CI): 1.11 (0.99, 1.25);  High HDP: 1.003 (0.795, 1.262);  Low HDP: 1.198 (0.953, 1.506);  Pint = 0.7768;  High SFP: 1.241 (0.967, 1.595);  Low SFP: 1.064 (0.834, 1.355);  Pint = 0.3048;  High AFP: 1.259 (0.982, 1.616);  Low AFP: 0.994 (0.775, 1.271);  Pint = 0.0857;  High TP: 1.26 (1.00, 1.59);  Low TP: 1.18 (0.934, 1.49);  Pint = 0.2241;  PM_10_:  Total OR (95%CI): 1.07 (0.96, 1.19);  High HDP: 0.995 (0.803, 1.231);  Low HDP: 1.14 (0.919, 1.415);  Pint = 0.7343;  High SFP: 1.199 (0.948, 1.52);  Low SFP: 0.999 (0.791, 1.258);  Pint = 0.2158;  High AFP: 1.163 (0.921, 1.473);  Low AFP: 0.995 (0.786, 1.259);  Pint = 0.2203;  High TP: 1.202 (0.964, 1.5);  Low TP: 1.102 (0.886, 1.371);  Pint = 0.2399;  SO_2_:  Total OR (95%CI): 1.17 (1.03, 1.33);  High HDP: 1.034 (0.798, 1.334);  Low HDP: 1.256 (0.968, 1.627);  Pint = 0.9451;  High SFP: 1.331 (0.998, 1.776);  Low SFP: 1.095 (0.841, 1.42);  Pint = 0.2818;  High AFP: 1.262 (0.961, 1.652);  Low AFP: 1.023 (0.781, 1.333);  Pint = 0.0986;  High TP: 1.234 (0.952, 1.596);  Low TP: 1.319 (1.007, 1.726);  Pint = 0.3353;  NO_2_:  Total OR (95%CI): 1.19 (1.06, 1.34);  High HDP: 1.084 (0.861, 1.361);  Low HDP: 1.216 (0.96, 1.544);  Pint = 0.911;  High SFP: 1.287 (0.993, 1.67);  Low SFP: 1.28 (1.019, 1.608);  Pint = 0.922;  High AFP: 1.412 (1.093, 1.831);  Low AFP: 1.067 (0.844, 1.345);  Pint = 0.024;  High TP: 1.163 (0.926, 1.46);  Low TP: 1.348 (1.043, 1.744);  Pint = 0.889;  CO:  Total OR (95%CI): 1.18 (1.05, 1.33);  High HDP: 1.112 (0.879, 1.403);  Low HDP: 1.185 (0.916, 1.532);  Pint = 0.582;  High SFP: 1.273 (0.971, 1.666);  Low SFP: 1.203 (0.949, 1.522);  Pint = 0.718;  High AFP: 1.355 (1.049, 1.757);  Low AFP: 1.046 (0.814, 1.336);  Pint = 0.042;  High TP: 1.186 (0.943, 1.492);  Low TP: 1.226 (0.946, 1.585);  Pint = 0.410;  O_3_:  Total OR (95%CI): 0.77 (0.68, 0.88);  High HDP: 0.832 (0.645, 1.07);  Low HDP: 0.751 (0.576, 0.976);  Pint = 0.662;  High SFP: 0.776 (0.58, 1.032);  Low SFP: 0.722 (0.555, 0.934);  Pint = 0.821;  High AFP: 0.714 (0.539, 0.94);  Low AFP: 0.736 (0.56, 0.963);  Pint = 0.337;  High TP: 0.755 (0.577, 0.982);  Low TP: 0.705 (0.537, 0.92);  Pint = 0.536; | Race; education level; family income; physical activity; family history of diabetes; parity; smoking and drinking status; the presence of hypertension/cardiac diseases, eclampsia/ polycystic ovarian syndrome, or depression or other chronic diseases; total energy intake; cooking behavior and change in residence. |
| Jin et al. (2024) | UK | 6 years | 56.64 y | Cohort | 257,534 | Omega-3 fatty acids | O_3_; | Depression | O_3_:  Total HR (95%CI): 1.23 (1.16, 1.30);  High omega-3 fatty acids: 0.91 (0.71, 1.17);  Low omega-3 fatty acids: 1.44 (1.10, 1.88);  Pint = 0.012; | Age, sex, ethnicity, Townsend deprivation, urbanicity, education, smoking status, alcohol intake frequency, FEV1, FVC, diagnosed COPD, diagnosed diabetes, and CVD status, and number of taken treatment/medication, PM_2.5_. |
| Li (b) et al. (2023) | UK | 9.7 y | 37-73 y | Cohort | 354,897 | FV; Oily fish; Red meat; Processed meat | PM_10_; PM_2.5_; NO_2_; NO_X_ | Depression | PM_10_:  Total HR (95%CI): 1.00 (0.92, 1.09);  High FV: 0.99 (0.90, 1.10);  Low FV: 0.96 (0.79, 1.16);  Pint = 0.955;  High oily fish: 1.05 (0.93, 1.18);  Low oily fish: 0.95 (0.84, 1.09);  Pint = 0.603;  High red meat: 1.00 (0.88, 1.14);  Low red meat: 0.96 (0.85, 1.08);  Pint = 0.456;  High processed meat: 1.10 (0.94, 1.29);  Low processed meat: 0.95 (0.86, 1.06);  Pint = 0.081;  PM_2.5_:  Total HR (95%CI): 1.16 (1.07, 1.26);  High FV: 1.16 (1.06, 1.28);  Low FV: 1.07 (0.90, 1.28);  Pint = 0.43;  High oily fish: 1.24 (1.11, 1.39);  Low oily fish: 1.08 (0.96, 1.22);  Pint = 0.794;  High red meat: 1.20 (1.06, 1.36);  Low red meat: 1.08 (0.96, 1.21);  Pint = 0.089;  High processed meat: 1.28 (1.11, 1.47);  Low processed meat: 1.10 (1.00, 1.22);  Pint = 0.022;  NO_2_:  Total HR (95%CI): 1.00 (0.98, 1.02);  High FV: 1.00 (0.97, 1.03);  Low FV: 0.97 (0.92, 1.03);  Pint = 0.882;  High oily fish: 1.03 (0.99, 1.06);  Low oily fish: 0.97 (0.94, 1.00);  Pint = 0.405;  High red meat: 1.01 (0.97, 1.05);  Low red meat: 0.98 (0.94, 1.01);  Pint = 0.059;  High processed meat: 1.02 (0.97, 1.06);  Low processed meat: 0.99 (0.96, 1.02);  Pint = 0.140;  NO_X_:  Total HR (95%CI): 1.02 (1.01, 1.05);  High FV: 1.02 (1.00, 1.05);  Low FV: 1.01 (0.96, 1.06);  Pint = 0.740;  High oily fish: 1.05 (1.02, 1.08);  Low oily fish: 0.99 (0.96, 1.03);  Pint = 0.247;  High red meat: 1.03 (1.00, 1.06);  Low red meat: 1.01 (0.98, 1.04);  Pint = 0.140;  High processed meat: 1.04 (1.00, 1.08);  Low processed meat: 1.02 (0.99, 1.04);  Pint = 0.154; | Age, sex, ethnicity, education level, employment status, household income, and Townsend deprivation index |
| Chen et al. (2022) | USA | 9 y | 65-80 y | Cohort | 7,183 | Folate; VB_12_;  VB_6_ | PM_2.5_ | Dementia | PM_2.5_:  Total HR (95%CI): 1.15 (0.88, 1.51);  High folate: 0.86 (0.59, 1.24);  Low folate: 1.53(1.07, 2.21);  Pint = 0.020;  High VB_12_: 0.90 (0.62, 1.30);  Low VB_12_: 1.47(1.02, 2.11);  Pint = 0.051;  High VB_6_: 0.91 (0.64, 1.30);  Low VB_6_: 1.50 (1.03, 2.19);  Pint = 0.046; | Age; race; US region; education; employment; family income; BMI; smoking status; alcohol consumption; physical activity; prior depression; randomization assignment in WHI trials; and histories of hypercholesterolemia, diabetes, hypertension, and cardiovascular diseases |
| Zhang et al. (2025) | UK | 11 y | 37−73 y | Cohort | 115,467 | Neurodegenerative Delay diet score (MIND diet); HDP; | PM_10_; PM_2.5_; NO_2_; NO_X_ | Neurodegenerative Diseases | PM_2.5_:  Total HR (95%CI): 1.21 (1.01, 1.45);  High MIND diet: 1.15 (0.92, 1.44);  Low MIND diet: 1.31 (1.03, 1.66);  Pint = 0.403;  High HDP: 1.51 (0.80, 2.84);  Low HDP: 1.21 (1.01, 1.45);  Pint = 0.502;  PM_10_:  Total HR (95%CI): 1.13 (0.99, 1.28);  High MIND diet: 1.08 (0.92, 1.26);  Low MIND diet: 1.20 (1.01, 1.44);  Pint = 0.322;  High HDP: 1.21 (0.75, 1.95);  Low HDP: 1.13 (0.99, 1.28);  Pint = 0.789;  NO_2_:  Total HR (95%CI): 1.14 (1.02,1.26);  High MIND diet: 1.06 (0.93, 1.20);  Low MIND diet: 1.24 (1.08, 1.42);  Pint = 0.056;  High HDP: 1.20 (0.85, 1.69);  Low HDP: 1.13 (1.02, 1.26);  Pint = 0.750;  NO_X_:  Total HR (95%CI): 1.07 (1.02, 1.13);  High MIND diet: 1.03 (0.96, 1.10);  Low MIND diet: 1.13 (1.05, 1.21);  Pint = 0.042;  High HDP: 1.07 (0.90, 1.29);  Low HDP: 1.07 (1.02, 1.14);  Pint = 0.997; | Age, sex, ethnicity, obesity, smoking status, physical activity, sleep duration, education, economic status, loneliness score, social isolation, annual household income, urbanity, Townsend deprivation index and family history of NDD |
| Zhu et al. (2022) | China | 5.6 y | 65-110 y | cohort | 6525 | Plant-based diet index (PDI) | PM_2.5_ | Cognitive function | PM_2.5_:  Total OR (95%CI): 1.10 (1.04, 1.15);  High PDI: 1.07(1.01, 1.14);  Low PDI: 1.13(1.05, 1.21);  Pint = 0.04; | Age, sex, urban/rural residence, education, occupation before age 60, financial status, social and leisure activity, smoking and drinking status, regular exercise, and geographic regions |
| Guo et al. (2022) | China | — | 30–79 y | Cross-sectional | 90,086 | Fat | PM_1_;  PM_2.5_; PM_10_; NO_2_ | MAFLD | PM_1_:  Total OR (95%CI): 1.13 (1.10, 1.17);  High fat: 1.16(1.12, 1.21);  Low fat: 1.08(1.04, 1.13);  Pint = 0.013;  PM_2.5_:  Total OR (95%CI): 1.29 (1.25, 1.34);  High fat: 1.38 (1.30, 1.46);  Low fat: 1.20 (1.13, 1.27);  Pint = 0.001;  PM_10_:  Total OR (95%CI): 1.11 (1.09, 1.14);  High fat: 1.16 (1.12, 1.21);  Low fat: 1.07 (1.05, 1.09);  Pint < 0.001;  NO_2_:  Total OR (95%CI): 1.15 (1.12, 1.17);  High fat: 1.17 (1.15, 1.20);  Low fat: 1.11 (1.06, 1.15);  Pint = 0.007; | Age, sex, ethnicity, education attainment, annual household income, study region, alcohol consumption, smoking status, second-hands smoke, low fruit and vegetable intake, physical activity, and indoor air pollution |
| Li (c) et al. (2022) | UK | 11.7 y | 40 -69 y | Cohort | 455,210 | Diet quality score (DQS) | NO_2_;  NO_X_ | Ulcerative colitis; Crohn’s disease | Ulcerative colitis:  NO_2_:  Total HR (95%CI): 1.12 (1.07, 1.19);  High DQS: 1.12 (1.04, 1.20);  Low DQS: 1.14 (1.05, 1.24);  Pint = 0.632;  NO_X_:  Total HR (95%CI): 1.07 (1.02, 1.12);  High DQS: 1.07 (1.01, 1.14);  Low DQS: 1.06 (0.99, 1.14);  Pint = 0.482;  Crohn’s disease:  NO_X_:  Total HR (95%CI): 0.98 (0.91, 1.05);  High DQS: 1.04 (0.94, 1.14);  Low DQS: 0.90 (0.80, 1.02);  Pint = 0.386; | Age, sex, ethnicity, household income, BMI, smoking status, healthy diet score, alcohol consumption, sedentary time, aspirin use, inflammatory disease and respiratory disease |

Abbreviations: AA: arachidonic acid; ALA: α-Linolenic acid; Anti-EDIP: anti-Empirical Dietary Inflammatory Pattern; Anti-PONDP: anti the pro-oxidative nutrients-derived dietary pattern; AODP: anti-oxidative dietary pattern; AONDP: anti-oxidative nutrients-derived dietary pattern; CO: carbon monoxide; COPD: chronic obstructive pulmonary disease; DASH: dietary approaches to stop hypertension; DHA: docosahexaenoic acid; DLP: dyslipidemia; DPA: docosapentaenoic acid; EPA: eicosapentaenoic acid; HALP: hypoalphalipo-proteinemia; HBLP: hyperbetalipo-proteinemia; HCH: hypercholesterolemia; HR: hazards ratio; HTG: hypertriglyceridemia; LA: linoleic acid; MAFLD: metabolic dysfunction-associated fatty liver disease; MED: mediterranean diet; NO_X_: nitrogen oxides; O_3_: ozone; PM: particulate matter; SO_2_: sulfur dioxide.

**Supplementary Table 4.** Dietary factors corresponding to diseases of various systems.

| **Disease systems** | **Dietary factors** | **Specific types of dietary factors included** | **Specific diseases included** |
| --- | --- | --- | --- |
| Cardiovascular diseases | HDPs | The Mediterranean diet; the Dietary Approaches to Stop Hypertension diet; Healthy plant-based dietary patterns; Overall diet quality scores; Balanced eating habit; Traditional southern pattern; Composite Dietary Antioxidant Index; | Heart failure; Hypertension; Stroke; Ventricular arrhythmias; |
|  | UDPs | Dietary inflammatory index; | Stroke; Ischemic heart disease; |
|  | Foods | Fruits; Vegetables; Grain; Meat, Fish; Dairy; Sugar-sweetened beverages; Legume; Fungus-based foods; | Hypertension; |
|  | Nutrients | Sodium; | Hypertension; |
| Chronic respiratory diseases | HDPs | Plant based dietary pattern; Mediterranean diet | COPD; Lung cancer; |
|  | UDPs | Dietary inflammatory index; | COPD; Lung cancer; |
|  | Foods | Fruits; | COPD; Lung cancer; |
|  | Nutrients | Omega-3 fatty acids; Omega-6 fatty acids; | Allergic rhinitis; COPD; Lung cancer; |
| Metabolic diseases | HDPs | The Mediterranean diet; The Dietary Approaches to Stop Hypertension diet; Anti-Empirical Dietary Inflammatory Pattern; Anti-oxidative dietary pattern; Anti the pro-oxidative nutrients-derived dietary pattern; Anti-oxidative nutrients-derived dietary pattern; The total healthy beverage score; Controlled low-calorie and low-fat diets; The Mini Dietary Assessment Index; The dietary diversity score; Healthy pattern; | Diabetes; Metabolic syndrome; |
|  | UDPs | Dietary inflammatory index; Empirical dietary inflammatory pattern score; Sweet foods pattern; Animal foods pattern; Traditional pattern; | Gestational diabetes mellitus; |
|  | Foods | Fruits; Vegetables; Meat; Sugar-sweetened beverages; | Obesity; Diabetes; Metabolic syndrome; Hypertriglyceridemia; Hypoalphalipo-proteinemia; Hyperbetalipo-proteinemia; Dyslipidemia; |
|  | Nutrients | Fat; Vitamin A; Vitamin D; Sodium; | Obesity; Diabetes; |
| Neuropsychiatric disorders | HDPs | Neurodegenerative Delay diet score; Healthy diet; Plant-based diet index; | Neurodegenerative diseases; Cognitive dysfunction; |
|  | Foods | Meat; | Depression; |
|  | Nutrients | Vitamin B; | Dementia; |
| Digestive disorders | HDPs | Diet quality score; | Crohn’s disease; Ulcerative colitis; |
|  | Nutrients | Fat; | Metabolic dysfunction-associated fatty liver disease; |

Abbreviations: COPD: chronic obstructive pulmonary disease. HDPs: healthy dietary patterns; UDPs: unhealthy dietary patterns.

**Supplementary Table 5.** Quality assessments of included cohort studies (N = 30).

| **Study** | **Selection** | | | | **Comparability** | **Outcome** | | | **Total**  **score** |
| --- | --- | --- | --- | --- | --- | --- | --- | --- | --- |
|  | **Exposed** | **Non-exposed** | **Ascertainment** | **Outcome** |  | **Assessment** | **Length of** | **Adequacy of** |  |
|  | **Cohort** | **Cohort** | **of exposure** | **of interest** |  | **of outcome** | **follow-up** | **follow-up** |  |
| Li (a) et al. (2022) | 0 | 1 | 1 | 1 | 2 | 0 | 1 | 1 | 7 |
| Andersen et al. (2011) | 1 | 1 | 1 | 1 | 2 | 1 | 1 | 1 | 9 |
| Chen et al. (2023) | 1 | 1 | 1 | 1 | 2 | 1 | 1 | 1 | 9 |
| Shin et al. (2023) | 1 | 1 | 1 | 1 | 2 | 1 | 1 | 0 | 8 |
| Chen et al. (2024) | 1 | 1 | 1 | 1 | 2 | 1 | 1 | 0 | 8 |
| Fan et al. (2024) | 1 | 1 | 1 | 1 | 2 | 1 | 1 | 1 | 9 |
| Margetaki et al. (2024) | 1 | 1 | 1 | 1 | 2 | 1 | 1 | 0 | 8 |
| Zheng et al. (2024) | 1 | 1 | 1 | 1 | 2 | 1 | 1 | 1 | 9 |
| Jin et al. (2024) | 1 | 1 | 1 | 1 | 2 | 1 | 1 | 1 | 9 |
| Zhu et al. (2024) | 1 | 1 | 1 | 1 | 1 | 1 | 1 | 0 | 7 |
| Ma et al. (2025) | 1 | 1 | 1 | 1 | 2 | 1 | 1 | 0 | 8 |
| Wang et al. (2025) | 1 | 1 | 1 | 1 | 1 | 0 | 1 | 0 | 6 |
| Fan et al. (2025) | 1 | 1 | 1 | 1 | 2 | 1 | 1 | 1 | 9 |
| Mishra et al. (2025) | 1 | 1 | 1 | 1 | 2 | 1 | 1 | 0 | 8 |
| Li (b) et al. (2022) | 1 | 1 | 1 | 1 | 2 | 1 | 1 | 0 | 8 |
| Guo et al. (2025) | 1 | 1 | 1 | 1 | 1 | 1 | 1 | 1 | 8 |
| Ole et al. (2011) | 1 | 1 | 1 | 1 | 1 | 1 | 1 | 1 | 8 |
| Li (a) et al. (2023) | 1 | 1 | 1 | 1 | 1 | 1 | 1 | 0 | 7 |
| Li (b) et al. (2023) | 1 | 1 | 1 | 1 | 1 | 1 | 1 | 1 | 8 |
| Chen et al. (2022) | 1 | 1 | 1 | 1 | 2 | 1 | 1 | 1 | 9 |
| **Study** | **Selection** | | | | **Comparability** | **Outcome** | | | **Total**  **score** |
|  | **Exposed** | **Non-exposed** | **Ascertainment** | **Outcome** |  | **Assessment** | **Length of** | **Adequacy of** |  |
|  | **Cohort** | **Cohort** | **of exposure** | **of interest** |  | **of outcome** | **follow-up** | **follow-up** |  |
| Zhang et al. (2016) | 1 | 1 | 1 | 1 | 1 | 1 | 1 | 1 | 8 |
| Lei et al. (2025) | 1 | 1 | 1 | 1 | 1 | 1 | 1 | 0 | 7 |
| Zhu et al. (2025) | 1 | 1 | 1 | 1 | 1 | 1 | 1 | 0 | 7 |
| Cheng et al. (2025) | 1 | 1 | 1 | 1 | 1 | 1 | 1 | 1 | 8 |
| Ji et al. (2025) | 1 | 1 | 1 | 1 | 1 | 1 | 1 | 0 | 8 |
| Xu (b) et al. (2021) | 1 | 1 | 1 | 1 | 1 | 0 | 1 | 0 | 6 |
| Li (c) et al. (2022) | 1 | 1 | 1 | 1 | 1 | 1 | 1 | 1 | 8 |
| Zhang et al. (2021) | 1 | 1 | 1 | 1 | 2 | 1 | 1 | 0 | 8 |
| Zhu et al. (2022) | 1 | 1 | 1 | 1 | 2 | 1 | 1 | 0 | 8 |
| Zhang et al. (2025) | 1 | 1 | 1 | 1 | 1 | 1 | 1 | 1 | 8 |

Note: The study quality was assessed according to an adapted form of the Newcastle Ottawa Quality (NOS). This scale awards a maximum of 9 points to each study: 4 for selection, 2 for comparability, and 3 for assessment of outcomes.

**Supplementary Table 6.** Quality assessments of included case-control studies (N = 1).

| **Study** | **Selection** | | | | **Compara-bility** | **Outcome** | | | **Total**  **score** |
| --- | --- | --- | --- | --- | --- | --- | --- | --- | --- |
|  | **Ascertainment**  **of cases** | **Representative-ness of cases** | **Selection of control** | **Ascertainment**  **of control** |  | **Ascertainment**  **of exposure** | **Same method of determination** | **Non-response rate** |  |
| Lamichhane et al. (2017) | 1 | 1 | 0 | 1 | 1 | 1 | 1 | 0 | 6 |

Note: The study quality was assessed according to an adapted form of the Newcastle Ottawa Quality (NOS). This scale awards a maximum of 9 points to each study: 4 for selection, 2 for comparability, and 3 for assessment of outcomes.

**Supplementary Table 7.** Quality assessments of included cross-sectional studies (N = 20).

| **Items** | Lin et al. (2017) | Zu et al. (2024) | Zhang et al. (2020) | Xu (a) et al. (2021) | Zhang et al. (2024) | Huang et al. (2023) | Zu et al. (2023) |
| --- | --- | --- | --- | --- | --- | --- | --- |
| Define the source of information | 1 | 1 | 1 | 1 | 1 | 1 | 1 |
| List inclusion and exclusion criteria | 1 | 1 | 1 | 1 | 1 | 1 | 1 |
| Indicate time period used for identifying patients; | 1 | 1 | 1 | 1 | 1 | 1 | 1 |
| Indicate whether or not subjects were consecutive if not population-based; | 1 | 1 | 1 | 1 | 1 | 1 | 1 |
| Indicate whether there is an evaluator’s subjective research component | 0 | 1 | 1 | 0 | 0 | 1 | 1 |
| Describe any assessments undertaken for quality assurance purposes | 0 | 0 | 1 | 0 | 0 | 0 | 0 |
| Explain any patient exclusions from analysis | 1 | 1 | 1 | 1 | 1 | 1 | 1 |
| Describe how confounding was assessed and/or controlled | 1 | 1 | 1 | 1 | 1 | 1 | 1 |
| Explain how missing data were handled in the analysis | 0 | 0 | 0 | 0 | 0 | 0 | 0 |
| Summarize patient response rates and completeness of data collection | 1 | 0 | 1 | 1 | 1 | 1 | 1 |
| Clarify what follow-up and the percentage of patients for which incomplete data | 0 | 1 | 1 | 0 | 0 | 0 | 0 |
| Total score | 7 | 8 | 10 | 7 | 7 | 8 | 8 |

| **Items** | Kwak et al.  (2023) | Guo et al. (2020) | Mao et al. (2020) | Li et al. (2019) | Yang et al. (2025) | Liu et al. (2020) | Yang (a) et al. (2018) |
| --- | --- | --- | --- | --- | --- | --- | --- |
| Define the source of information | 1 | 1 | 1 | 1 | 1 | 1 | 1 |
| List inclusion and exclusion criteria | 1 | 1 | 1 | 1 | 1 | 1 | 1 |
| Indicate time period used for identifying patients; | 1 | 1 | 1 | 1 | 1 | 1 | 1 |
| Indicate whether or not subjects were consecutive if not population-based; | 1 | 1 | 1 | 1 | 1 | 1 | 1 |
| Indicate whether there is an evaluator's subjective research component | 1 | 1 | 1 | 1 | 1 | 1 | 0 |
| Describe any assessments undertaken for quality assurance purposes | 1 | 1 | 0 | 1 | 1 | 1 | 0 |
| Explain any patient exclusions from analysis | 1 | 1 | 1 | 1 | 1 | 1 | 1 |
| Describe how confounding was assessed and/or controlled | 1 | 1 | 1 | 1 | 1 | 1 | 1 |
| Explain how missing data were handled in the analysis | 1 | 0 | 0 | 0 | 1 | 0 | 0 |
| Summarize patient response rates and completeness of data collection | 1 | 1 | 1 | 1 | 1 | 1 | 1 |
| Clarify what follow-up and the percentage of patients for which incomplete data | 0 | 0 | 0 | 0 | 1 | 0 | 0 |
| Total score | 10 | 9 | 8 | 9 | 11 | 9 | 7 |

| **Items** | Cao et al. (2024) | Wang et al. (2022) | Guo et al. (2022) | Yang (b) et al. (2018) | Zheng et el. (2022) | Cai et al. (2023) |
| --- | --- | --- | --- | --- | --- | --- |
| Define the source of information | 1 | 1 | 1 | 1 | 1 | 1 |
| List inclusion and exclusion criteria | 1 | 1 | 1 | 1 | 1 | 1 |
| Indicate time period used for identifying patients; | 1 | 1 | 1 | 1 | 1 | 1 |
| Indicate whether or not subjects were consecutive if not population-based; | 1 | 1 | 1 | 1 | 1 | 1 |
| Indicate whether there is an evaluator's subjective research component | 1 | 0 | 1 | 0 | 0 | 0 |
| Describe any assessments undertaken for quality assurance purposes | 0 | 0 | 1 | 1 | 0 | 0 |
| Explain any patient exclusions from analysis | 1 | 1 | 1 | 1 | 1 | 1 |
| Describe how confounding was assessed and/or controlled | 1 | 1 | 1 | 1 | 1 | 1 |
| Explain how missing data were handled in the analysis | 0 | 0 | 1 | 0 | 0 | 0 |
| Summarize patient response rates and completeness of data collection | 1 | 1 | 1 | 1 | 1 | 1 |
| Clarify what follow-up and the percentage of patients for which incomplete data | 0 | 0 | 0 | 0 | 0 | 0 |
| Total score | 8 | 7 | 10 | 8 | 7 | 7 |

Note: The study quality was assessed according to an adapted form of the Agency for Healthcare Research and Quality (AHRQ). This scale awards a maximum of 11 points to each study.

**Supplementary** **Table 8.** The modifying effect of the dietary item in a single study.

| **Author, year** | **Air pollutants** | **Dietary factors** | **Effect value of high intake** | **Effect value of Low intake** | ***P* for interaction** | **Outcome** |
| --- | --- | --- | --- | --- | --- | --- |
| Guo et al. (2025) | SO_2_ | UDP | 1.07 (1.02, 1.13) | 1.05 (1.00, 1.11) | 0.420 | Stroke |
| Guo et al. (2025) | CO | UDP | 1.00 (1.00, 1.00) | 1.00 (1.00, 1.00) | 0.390 | Stroke |
| **Guo et al. (2025)** | **SO_2_** | **UDP** | **1.06 (1.03, 1.09)** | **1.02 (0.99, 1.05)** | **0.005** | **Ischemic heart disease** |
| Guo et al. (2025) | CO | UDP | 1.00 (1.00, 1.00) | 1.00 (1.00, 1.00) | 0.900 | Ischemic heart disease |
| Cheng et al. (2025) | NO_X_ | HDP | 1.06 (1.01, 1.12) | 1.10 (1.01, 1.20) | 0.330 | Ventricular arrhythmias |
| Cheng et al. (2025) | NO_X_ | HDP | 1.04 (0.99, 1.09) | 1.09 (1.01, 1.18) | 0.130 | Ventricular arrhythmias |
| **Ji et al. (2025)** | **O_3_** | **HDP** | **1.39 (1.39, 1.40)** | **1.65 (1.62, 1.68)** | **< 0.001** | **CVDS** |
| **Ji et al. (2025)** | **CO** | **HDP** | **1.01 (1.00, 1.01)** | **1.02 (1.02, 1.02)** | **< 0.001** | **CVDS** |
| Huang et al. (2023) | PM | Legumes and nuts | 1.09 (1.01, 1.17) | 1.15 (1.06, 1.24) | 0.260 | Hypertension |
| Zhang et al. (2020) | SO_2_ | Vegetables | 1.12 (0.77, 1.62) | 1.34 (0.91, 1.95) | 0.770 | Hypertension |
| Zhang et al. (2020) | SO_2_ | Fruits | 1.20 (0.85, 1.71) | 1.18 (0.79, 1.75) | 0.340 | Hypertension |
| Zhang et al. (2020) | SO_2_ | Meat | 1.81 (1.14, 2.89) | 1.61 (1.24, 2.08) | 0.440 | Hypertension |
| Zhang et al. (2020) | SO_2_ | Fish | 1.30 (0.89, 1.90) | 1.19 (0.83, 1.70) | 0.760 | Hypertension |
| Zhang et al. (2020) | SO_2_ | Sugar-sweetened beverages | 1.06 (0.74, 1.51) | 1.35 (0.92, 1.98) | 0.320 | Hypertension |
| Zhang et al. (2020) | SO_2_ | Milk | 1.57 (1.17, 2.10) | 1.89 (1.31, 2.75) | 0.350 | Hypertension |
| Zhang et al. (2020) | SO_2_ | Grain | 1.08 (0.75, 1.55) | 1.33 (0.90, 1.98) | 0.340 | Hypertension |
| Zhang et al. (2020) | SO_2_ | Soybean products | 1.34 (0.90, 1.97) | 1.28 (0.69, 2.37) | 0.160 | Hypertension |
| Zhang et al. (2020) | O_3_ | Vegetables | 1.13 (0.76, 1.67) | 1.36 (0.96, 1.91) | 0.560 | Hypertension |
| Zhang et al. (2020) | O_3_ | Fruits | 1.15 (0.80, 1.65) | 1.39 (0.97, 2.00) | 0.160 | Hypertension |
| Zhang et al. (2020) | O_3_ | Meat | 1.14 (0.74, 1.74) | 1.33 (0.97, 1.82) | 0.920 | Hypertension |
| Zhang et al. (2020) | O_3_ | Fish | 1.34 (0.95, 1.89) | 1.22 (0.84, 1.75) | 0.790 | Hypertension |
| Zhang et al. (2020) | O_3_ | Sugar-sweetened beverages | 1.56 (1.22, 1.99) | 1.41 (1.10, 1.81) | 0.130 | Hypertension |
| Zhang et al. (2020) | O_3_ | Milk | 0.96 (0.62, 1.48) | 1.64 (1.16, 2.32) | 0.900 | Hypertension |
| Zhang et al. (2020) | O_3_ | Grain | 1.27 (0.90, 1.78) | 1.22 (0.81, 1.82) | 0.260 | Hypertension |
| Zhang et al. (2020) | O_3_ | Soybean products | 1.52 (1.06, 2.17) | 1.07 (0.74, 1.54) | 0.050 | Hypertension |
| **Li et al. (2019)** | **PM** | **Fat** | **1.08 (1.06, 1.10)** | **1.03 (1.02, 1.05)** | **< 0.001** | **Hypertension** |
| Guo et al. (2025) | SO_2_ | UDP | 1.06 (1.02, 1.10) | 1.07 (1.02, 1.13) | 0.690 | COPD |
| Guo et al. (2025) | CO | UDP | 1.00 (1.00, 1.00) | 1.00 (1.00, 1.00) | 0.130 | COPD |
| Guo et al. (2025) | SO_2_ | UDP | 0.95 (0.87, 1.05) | 0.96 (0.85, 1.08) | 0.730 | Lung cancer |
| Guo et al. (2025) | CO | UDP | 1.00 (1.00, 1.01) | 1.00 (1.00, 1.01) | 0.970 | Lung cancer |
| Yang et al. (2025) | SO_2_ | HDP | 1.21 (1.14, 1.28) | 1.18 (1.10, 1.28) | 0.170 | Lung cancer |
| Lamichhane et al. (2017) | PM | Fruits | 1.01 (0.77, 1.31) | 1.44 (1.06, 1.96) | 0.200 | Lung cancer |
| Chen et al. (2023) | NO_X_ | Omega-6 fatty acids | 1.04 (0.99, 1.10) | 1.05 (1.00, 1.10) | — | Lung cancer |
| Chen et al. (2023) | NO_X_ | Omega-6 fatty acids | 1.06 (0.93, 1.20) | 1.07 (0.96, 1.20) | — | Lung cancer |
| Yang et al. (2018) (b) | SO_2_ | Sugar-sweetened soft drink | 1.19 (1.10, 1.25) | 1.11 (1.08, 1.14) | > 0.050 | Metabolic syndrome |
| Yang et al. (2018) (b) | NO_X_ | Sugar-sweetened soft drink | 1.56 (1.40, 1.74) | 1.36 (1.27, 1.45) | > 0.050 | Metabolic syndrome |
| Yang et al. (2018) (b) | O_3_ | Sugar-sweetened soft drink | 1.20 (1.12, 1.27) | 1.10 (1.07, 1.13) | > 0.050 | Metabolic syndrome |
| Yang et al. (2018) (b) | O_3_ | HDP | 1.11 (1.05, 1.17) | 1.10 (1.07, 1.14) | > 0.050 | Metabolic syndrome |
| Shin et al. (2023) | PM | Milk | 1.44 (0.93, 2.22) | 1.48 (1.02, 2.16) | 0.660 | Diabetes |
| Shin et al. (2023) | NO_X_ | Milk | 1.20 (0.94, 1.52) | 1.39 (1.15, 1.67) | 0.220 | Diabetes |
| Wang et al. (2022) | O_3_ | Fruits | 1.08 (1.02, 1.13) | 1.06 (0.97, 1.15) | 0.750 | Metabolic syndrome |
| Wang et al. (2022) | SO_2_ | Fruits | 0.87 (0.76, 0.98) | 0.92 (0.72, 1.13) | 0.680 | Metabolic syndrome |
| Zheng et al. (2022) | PM | Grain | 1.09 (0.90, 1.34) | 1.32 (1.08, 1.62) | 0.710 | Metabolic syndrome |
| Zheng et al. (2022) | PM | Grain | 1.33 (1.11, 1.61) | 1.38 (1.14, 1.68) | 0.900 | Hypertriglyceridemia |
| Li (b) et al. (2023) | PM | Fruit and vegetable | 1.16 (1.06, 1.28) | 1.07 (0.90, 1.28) | 0.430 | Depression |
| Li (b) et al. (2023) | PM | Oily fish | 1.24 (1.11, 1.39) | 1.08 (0.96, 1.22) | 0.790 | Depression |
| Li (b) et al. (2023) | PM | Fruit and vegetable | 0.99 (0.90, 1.10) | 0.96 (0.79, 1.16) | 0.960 | Depression |
| Li (b) et al. (2023) | PM | Oily fish | 1.05 (0.93, 1.18) | 0.95 (0.84, 1.09) | 0.600 | Depression |
| Li (b) et al. (2023) | NO_X_ | Fruit and vegetable | 1.00 (0.97, 1.03) | 0.97 (0.92, 1.03) | 0.880 | Depression |
| Li (b) et al. (2023) | NO_X_ | Oily fish | 1.03 (0.99, 1.06) | 0.97 (0.94, 1.00) | 0.410 | Depression |
| Li (b) et al. (2023) | NO_X_ | Fruit and vegetable | 1.02 (1.00, 1.05) | 1.01 (0.96, 1.06) | 0.740 | Depression |
| Li (b) et al. (2023) | NO_X_ | Oily fish | 1.05 (1.02, 1.08) | 0.99 (0.96, 1.03) | 0.250 | Depression |
| **Jin et al. (2024)** | **O_3_** | **Omega-3 fatty acids** | **0.91 (0.71, 1.17)** | **1.44 (1.10, 1.88)** | **0.012** | **Depression** |
| **Guo et al. (2022)** | **NO_X_** | **Fat** | **1.17 (1.15, 1.20)** | **1.11 (1.06, 1.15)** | **0.007** | **Metabolic dysfunction-associated fatty liver disease** |

Abbreviations: CI: confidence interval; CO: carbon monoxide; COPD: chronic obstructive pulmonary disease; CVDs: cardiovascular diseases; HDP: healthy dietary patterns; NO_X_: nitrogen oxides; O_3_: ozone; PM: particulate matter; SO_2_: sulfur dioxide; UDP: unhealthy dietary patterns.
